# Regulatory T-Cells at the Interface between Human Host and Pathogens in Infectious Diseases and Vaccination

**DOI:** 10.3389/fimmu.2015.00217

**Published:** 2015-05-11

**Authors:** Mardi C. Boer, Simone A. Joosten, Tom H. M. Ottenhoff

**Affiliations:** ^1^Department of Infectious Diseases, Leiden University Medical Center, Leiden, Netherlands

**Keywords:** regulatory T-cells, human regulatory T-cells, infection, vaccination, pathogens, tuberculosis, leprosy, BCG

## Abstract

Regulatory T-cells (Tregs) act at the interface of host and pathogen interactions in human infectious diseases. Tregs are induced by a wide range of pathogens, but distinct effects of Tregs have been demonstrated for different pathogens and in different stages of infection. Moreover, Tregs that are induced by a specific pathogen may non-specifically suppress immunity against other microbes and parasites. Thus, Treg effects need to be assessed not only in homologous but also in heterologous infections and vaccinations. Though Tregs protect the human host against excessive inflammation, they probably also increase the risk of pathogen persistence and chronic disease, and the possibility of disease reactivation later in life. *Mycobacterium leprae* and *Mycobacterium tuberculosis*, causing leprosy and tuberculosis, respectively, are among the most ancient microbes known to mankind, and are master manipulators of the immune system toward tolerance and pathogen persistence. The majority of mycobacterial infections occur in settings co-endemic for viral, parasitic, and (other) bacterial coinfections. In this paper, we discuss recent insights in the activation and activity of Tregs in human infectious diseases, with emphasis on early, late, and non-specific effects in disease, coinfections, and vaccination. We highlight mycobacterial infections as important models of modulation of host responses and vaccine-induced immunity by Tregs.

## Introduction

A myriad of innate and adaptive immune regulatory cells is induced upon infection, including cells of different lineages: regulatory-like macrophages, dendritic cells (DCs), NKT-cells, T-cells, B-cells, neutrophils, and mesenchymal stem cells. During the last decade, many reports have described the role of regulatory T-cells (Tregs) in infectious diseases and following vaccination. In infectious diseases, Tregs play a dual role: they benefit the host by limiting immune-mediated pathology and also facilitate chronic pathogen persistence by reducing effector immunity and clearance of infection (1). During acute infection, the beneficial role of Tregs seems to predominate, by regulating leukocyte in- and efflux into lymph nodes (LN) and infected sites, suppression of proliferation of infected cells, and favoring memory formation by increasing the time window of antigen availability.

Regulatory T-cells can be induced either in an antigen- and T-cell receptor (TCR)-dependent or in an antigen- and TCR-independent manner (2, 3). Specificity for self- or pathogen-derived antigens (or dual-specificity) was originally used to divide Treg populations into “natural” resp. “adaptive” Tregs, but it was recently recommended to denote Treg populations by place of induction: “thymus derived” or “peripherally derived,” or when the origin is unclear “Foxp3^+^ Treg cell” (4). Designations of human Tregs are, however, complicated by the fact that, unlike murine Tregs, unique markers are lacking. In addition, non-Treg populations can express “Treg markers” such as Foxp3 and CD25 upon activation; therefore, human Tregs are preferably defined by multiple regulatory markers and/or by demonstrating suppressive activity (5). Human CD8^+^ Tregs have been studied much less than CD4^+^ Tregs (5), even though they were among the first described “suppressor cells,” especially in mycobacterial infections (6, 7). The relative lack in studies on human CD8^+^ Tregs is possibly the result of technical difficulties in isolating and assessing functions of CD8^+^ T-cells (8). Notwithstanding, CD8^+^ Tregs are re-emerging as important players in general, including in human infectious disease and following vaccination (5).

Once activated, Tregs can suppress pro-inflammatory cells through several mechanisms that are adaptable to the local environment (9). These mechanisms can mostly be divided into inhibitory cytokine production (either membrane-bound or by their release in the pericellular environment), suppression by cytolysis, metabolic disruption of pro-inflammatory cells, modulation of antigen-presenting cells (APCs), and the activity of certain Treg membrane expressed molecules (see below) (10). These mechanisms indeed support the concept that antigen specifically induced Tregs can cross-suppress also other cells irrespective of the presence of their cognate antigen or specificity, e.g., through the secretion of cytokines (5). This “bystander” or heterologous suppression can compromise immunity toward unrelated pathogens, as has been described for coinfection by helminths in diseases such as malaria and tuberculosis (TB) (11). Helminth coinfections can also impair the immunogenicity of vaccines such as (oral) cholera vaccination and (intradermal) BCG (*Mycobacterium bovis* bacillus Calmette–Guérin) and tetanus vaccination (12). Several Treg-expressed molecular markers have now been implicated directly in mediating suppression, such as cytotoxic T-lymphocyte-associated antigen 4 (CTLA-4), which modulates APCs via its ligands CD80 and CD86. Tregs were shown to use trans-endocytosis of CD80 and CD86, followed by their intracellular degradation, thereby relatively depleting the APC’s expression of essential co-stimulatory receptors for T-cell CD28 ligation (13). In addition, the ecto-enzyme CD39 (E-NTPDase1), which is a relatively recently discovered Treg marker, exerts its suppressive effects through breakdown of adenosine triphosphate (ATP) (14).

In this paper, we will discuss the induction of Tregs (both specific and non-specific) by various pathogens as well as the functional implications of CD4^+^ and CD8^+^ Tregs in acute vs. chronic infectious diseases. We will discuss the role of Tregs in coinfections and highlight in particular infections with *M. leprae* and *M. tuberculosis* (Mtb), which are master manipulators of the human innate and adaptive immune response through the induction of regulatory circuits. We will discuss how the balance of pro- vs. anti-inflammatory responses could ultimately regulate pathogen persistence, and impact on the development of active vs. latent or reactivation of disease. We will also discuss the impact of Tregs on diagnosis and treatment of TB, as well as their possible impact on vaccination against TB.

## Mechanisms of Treg Induction by Pathogens

As a first line of host–defense against infection, the activation of innate immune cells through pattern recognition receptors (PRRs), such as Toll-like receptors (TLRs), lectin receptors, retinoic acid-inducible gene (RIG) receptors, scavenger, and phagocytic receptors, activates these cells to phagocytose and process the pathogen, after which they migrate to the draining lymph node (DLN) and present antigen to prime naïve T-cells. These cells then can differentiate into various classes of T-helper cells (Th), cytotoxic T-cells, or Tregs. Further activation and differentiation signals are provided to the T-cells upon migration into the infected tissue; these signals originate from other T-cells, activated tissue-resident APCs, or even directly from the pathogen (see below). Tissue-resident, circulating, and migrating APCs comprised heterogeneous populations, and the activation of APCs can lead to the induction of pro-inflammatory or regulatory, homeostatic T-cell responses (15): for example, pro-inflammatory human type-1 macrophages promote Th1-immunity and are characterized by IL-23 production and secretion of IL-12 after IFNγ stimulation, whereas type-2 macrophages poorly express co-stimulatory molecules, produce IL-10, and induce Tregs (16, 17).

Modulation of macrophages and DCs toward tolerogenic subsets has been described for various pathogens: after *in vitro* treatment of human DCs with Japanese encephalitis virus or Mtb, DCs upregulated the inhibitory receptor PD-L1, which induced the expansion of Tregs through PD-1 ligation (18–20). These effects were mediated by the Mtb-derived protein Acr (HspX Rv2031c), which is expressed during latency: Acr induced expression of PD-L1, TIM3, IDO, and IL-10 by murine DCs and promoted the induction of CD4^+^CD25^+^Foxp3^+^ T-cells (21). Furthermore, APCs can be modulated through alterations in (pericellular) purinergic pathways: extracellular ATP, a pro-inflammatory danger signal, which activates the killing of Mtb in macrophages, is rapidly hydrolyzed to AMP by CD39, which is expressed by various regulatory cells (14). The degradation of ATP to AMP in the microenvironment was accompanied by a switch in macrophage gene expression from type 1 toward type 2, and Mtb infection actively upregulated expression of the adenosine A2A receptor on macrophages (22). This receptor has been described as a major immunosuppressive immune cell adenosine receptor acting through elevation of cAMP (23), and its expression on macrophages was central to M2-like polarization after Mtb infection (22). Other cell types acting as APCs were demonstrated to contribute to Treg induction: both hepatitis C virus (HCV)-infected hepatocytes and *H. pylori*-infected gastric epithelial cells directly induced Tregs through production of TGF-β (24, 25).

Regulatory T-cells can also be induced directly through pathogen-derived components. This has been demonstrated in several murine studies: zwitterionic capsular polysaccharides from *S. pneumoniae* induced CD8^+^CD28^−^ Tregs that were CD122^LO^CTLA-4^+^CD39^+^, synthesized IL-10 and TGF-β, and exhibited suppressive activity. This induction was independent of APCs and involved direct crosslinking of the TCR (26). In another murine study, proteins secreted by *H. polygyrus* induced Foxp3^+^ T-cells through ligation of the TGF-β-receptor (27). The herpes virus entry mediator HVEM, a binding site for viral glycoprotein HSVgD, is upregulated on murine CD4^+^Foxp3^+^ Tregs after HSV-1 infection, and activation of this receptor led to preferential expansion of Tregs (28). In the human situation, CD4^+^CD25^+^ Tregs exhibited extended survival and increased suppressive capacity after binding HIVgp120 (29).

The preferential expression of TLRs, such as TLR2, on Tregs as compared to “conventional” T-cells has been reviewed by Sutmuller and colleagues (2). A large variety of TLR2 ligands have been described in bacteria, including Mtb (30). Mtb-induced TLR-signaling in APCs leads to inhibition of the MHC-II transactivator-gene CIITA, thereby decreasing expression of MHC-II and antigen presentation (30). During chronic Mtb infection, prolonged TLR2 signaling (e.g., through the 19kD lipoprotein) can lead to suppressive cytokine production (31) and recruitment of CD4^+^ Tregs to the lung (32). A role for TLR-mediated Treg induction has also been described in murine malaria: murine *Plasmodium*-activated DCs induced Tregs through TLR9, and TLR9^(−/−)^ mice had impaired activation of Tregs, associated with a partial resistance to lethal infection (33). Other factors in the local environment vital for the expansion and function of Tregs include changes in metabolism (34), endothelial cytokine (IL-33) production and cytokine balance (IL-23:IL-33 ratio) (35), and metabolite products from commensal microbiota (36, 37). Thus, specific pathogen components can skew toward Treg phenotype or function. The significance of these Tregs for the disease process, concomitant diseases, and vaccinations will be discussed further below.

## The Impact of Tregs in Infectious Diseases

### Viral infections: Acute vs. chronic infectious disease

**Figure 1:**
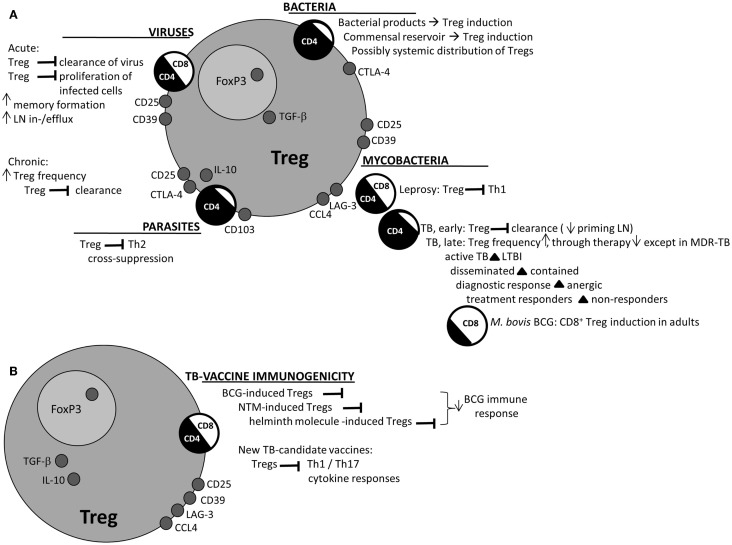
**Main effects of Tregs as described for various pathogens**. **(A)** Various Treg-mediated effects have been described for the various classes of pathogens; early vs. late, and heterologous suppression, are described in several taxonomies. Prominent features are noted, as well as prominent Treg markers for the various pathogens. Circles “CD4/CD8” depict the scale in reports of either CD4^+^ or CD8^+^ Tregs in literature for the various classes. **(B)** Treg effects on TB-vaccine immunogenicity are displayed in a similar fashion. BCG immunogenicity may be decreased by or inversely related to BCG-induced Tregs, or may be suppressed by heterogenic Tregs. Treg induction has also been described in various TB-vaccine candidate trials. BCG = *Mycobacterium bovis* bacillus Calmette–Guérin; CCL4 = CC chemokine ligand 4; CTLA-4 = cytotoxic T-lymphocyte-associated antigen 4; LAG-3 = lymphocyte activation gene-3; LN = lymph node; LTBI = latent tuberculosis infection; MDR-TB = multi-drug-resistant tuberculosis; NTM = non-tuberculous mycobacteria; TB = tuberculosis; Treg = regulatory T-cell.

Regulatory T-cells have been found after retrovirus-, RNA virus-, and DNA virus infection in mice and humans [reviewed in Ref. (3); Figure 1A]. Various CD4^+^ and CD8^+^ Treg subsets have been identified (38), but mostly in chronic viral infection. Yet, in hepatitis A virus infection – an acute inflammatory disease, usually followed by pathogen clearance – hepatitis A virus bound to its cellular receptor (HAVCR1), which is expressed on Tregs, which resulted in inhibited Treg function and inflammation (39). By contrast, in acute dengue fever, Treg function and the suppression of vasoactive cytokine release were similar in acutely infected and recovered patients, such that in this case, the disproportionate activation of pro-inflammatory cells and cytokines often found in dengue fever was not explained by acute phase Treg malfunction (40). Thus, blockade of Tregs in acute viral infection could assist in pathogen clearance, at the cost of temporary hyper-inflammation, but not all (pathological) hyper-inflammation is associated with Treg hypo-functionality. On the other side, Tregs could also benefit the host during acute infection: first, Treg depletion in murine herpes simplex infection increased LN levels of IFN-α and -γ, but infection-site-associated IFNγ was decreased, and the arrival of DCs, NK cells, and T-cells at the infected lesion was delayed (41), pointing to a role for Tregs in promoting LN in- and efflux of pro-inflammatory cells (42). Second, Tregs may suppress infected cell proliferation at the mucosal point-of-entry to a level where infection cannot be established, which was suggested as a protective mechanism in early HIV infection (43, 44). Third, Tregs were vital in allowing memory formation through promoting antigen persistence, as was recently demonstrated in a murine West Nile virus infection model (45).

The role of human Tregs in chronic viral infection has been more extensively delineated. A meta-analysis of 12 studies demonstrated increased CD4^+^ Treg frequencies in chronic hepatitis B virus (HBV) infection compared to both acute infection and healthy controls, revealing a strong association of Tregs with disease progression, viral load, absence of therapy response, and risk of hepatocellular carcinoma (46). In chronic HCV infection, the contribution of Tregs to low inflammatory CD4^+^ and CD8^+^ T-cell responses has been described (47, 48). Tregs were recruited to the liver through the Treg-attracting chemokines CCL17 and CCL22 (49), thereby promoting pathogen persistence. It has been argued, however, that Tregs may also be functional in limiting HCV-induced liver damage (48).

In chronic HIV infection, CD4^+^ Tregs were relatively increased in the mucosa and in the circulation compared to healthy controls, but the Treg-mediated effects on anti-HIV immune responses remain a matter of debate (50). CD4^+^ Tregs decreased HIV replication in T-cells *in vitro* through CD39-mediated ectonucleotide shifts and by transfer of cAMP through gap junctions formed with conventional T-cells (43). Tregs inhibited spreading of virus from DCs to T-cells through interfering with the immunological synapse (51). In another study, blocking of CD39 by monoclonal antibodies (mAbs) restored cytokine production by HIV-gag-stimulated CD8^+^ T-cells (52). Indeed, the relative frequency of CD4^+^CD39^+^ Tregs positively correlated with HIV viral load and disease progression in infected individuals (53). These different effects of Tregs could be explained by differentiating between acute and chronic infection, as argued in Ref. (50): control of viral replication by CD4^+^(CD39^+^) Tregs may be important early after infection with a limited number of infected cells (relatively high Treg: T-effector ratio), yet during chronic infection Tregs may not be able to suppress proliferation of all infected cells, and potentially become more detrimental due to dampening anti-HIV responses. This points to the need for more detailed analyses of Treg functions in acute vs. chronic (hyper-) inflammation.

### Bacterial infections: Reservoirs for Treg induction

Early vs. late effects of Tregs in bacterial infection were elegantly described in a mouse model of *Salmonella (Salmonella enterica* serotype Typhimurium): Tregs suppressed early protective immunity, thereby allowing for establishment of infection, yet clearance of infection at later time points corresponded with a decrease in Treg suppressive capacity (54). After acute infection, Treg-mediated failure to completely eradicate *Salmonella* may thus lead to a carrier state of persistent asymptomatic infection, resulting in a reservoir for shedding of pathogens into the environment and further infection [reviewed in Ref. (55)]. A carrier state of *Streptococcus pneumonia* in the nasopharynx was associated with increased TGF-β levels from nasal washes in humans, and TGF-β was shown to lead to Treg expansion in *in vitro* murine experiments (56). In *Helicobacter pylori* infection, a carrier state can last for life; and several studies have described the ability of *Helicobacter pylori* to induce Tregs. These Tregs were found in the circulation as well as in the gastric mucosa of both infected children and adults, and though Tregs initially can limit inflammation and therefore probably gastric ulceration, pathogen persistence could, on the other hand, lead to chronic inflammation and tumor induction (57) [reviewed in Ref. (58)].

Increasing attention has been drawn to the interplay of the immune system with non-pathogenic commensal microbiota in the intestine. Tregs can be induced by commensal microbiota, as has been demonstrated in multiple murine studies: butyrate, a metabolite from commensals, potently induced Tregs in the intestine (36, 37), possibly through butyrate-mediated enhanced histone H3 acetylation in the FOXP3 promoter (37). Polysaccharide A (PSA) from *B. fragilis* induced conversion of T-cells into Tregs, and cured experimental colitis (59). The CNRZ327-component from *Lactobacillus delbrueckii* induced regulatory responses in colonic tissue, but importantly also in cecal LNs and the spleen, pointing to systemic distribution of these microbiota-induced Tregs (60).

Raising mice in germ-free conditions decreased the number of Tregs in the gut, but the number of cutaneous Tregs was increased, possibly through loss of inhibition by pro-inflammatory cells (61). In any case, data on activation of Tregs by skin commensals is also emerging (61), and these Tregs induced by skin microbiota may modulate systemic inflammatory responses (61). As recently reviewed (62), increasing evidence reveals resident microbiota in the lungs. Though relatively low in bacterial biomass compared to the microbiota of the skin and the intestinal mucosa, these microorganisms are present in healthy lungs as they are at other mucosal surfaces, and probably differ in composition between healthy individuals and individuals with (pulmonary) disease (62).

Clearly, mucosal surfaces are the primary sites both for pathogenic and commensal microbiota; and induction of Tregs – within the myriad of innate and adaptive cells – has been described for both. Further research should elucidate local and systemic effects of Tregs induced at barrier sites in human studies, and whether systemic effects of Tregs induced by (non-pathogenic) commensals are to be expected (Figure 1A).

### Parasitic infections: Suppression across boundaries

Murine *Leishmaniasis* models have been pivotal in demonstrating Tregs at the site of (parasitic) infection: antigen-specific CD4^+^CD25^+^ Tregs were present at the site of chronic *Leishmania major* infection (63) and retention at the infection site was dependent on expression of CD103 by CD4^+^CD25^+^ Tregs (64). In this model, the impact of Tregs on establishment of chronic infection and reactivation of disease was elegantly demonstrated: after pathogen clearance, *Leishmania* super-infection led to reactivation of disease and increased Treg numbers at the primary site. Also, adoptive transfer of Tregs from infected mice into chronically infected mice caused reactivation of disease (65). Mechanisms of suppression included IL-10 production by Tregs as well as other mechanisms (66). In another study, Foxp3-negative cells were the major producers of IL-10, and anti-IL-10R mAb treatment decreased parasite burden to a greater extent compared to anti-CD25-mAb treatment (67). In humans, functionally suppressive CD4^+^CD25^+^ Tregs have been isolated from cutaneous leishmaniasis (skin) lesions (68); and FOXP3 mRNA levels in skin lesions were increased in chronic compared to acute *Leishmania major* infection (69). Also in *Leishmania guyanensis*-induced skin lesions, FOXP3 mRNA levels were significantly higher in chronic compared to acute patients, though in both cases Tregs isolated from these lesions displayed suppressive activity *in vitro* (70). Importantly, IL-10 and FOXP3 mRNA expression in *Leishmania guyanensis*-infected skin lesions were associated with unresponsiveness to treatment (71).

Several studies have reported increased Treg frequencies in *Plasmodium falciparum-*infected individuals compared to asymptomatic or uninfected controls (72); furthermore in patients with clinically severe malaria, the frequency of CD4^+^CD25^+^Foxp3^+^CD127^LO^ Treg cells correlated with levels of parasitemia and total parasite biomass (73). Tregs were associated with risk of malaria disease: reduced expression of CTLA-4 and FOXP3 was found in Fulani, an ethnic group in Burkina Faso relatively resistant to *P. falciparum* compared to Mossi (a different ethnic group from the same region) (74). Proliferative PBMC responses to malaria antigens from Mossi were increased following CD25^+^-depletion, but those from Fulani were not (74). In Kenyan adults with natural immunity to malaria, CD4^+^CD25^HI^ T-cell frequency at enrollment was associated with the risk of developing clinical malaria during follow-up (75).

Many helminth parasitic infections steer immunity toward Th2 and T-regulatory responses (12); and murine data indicate that immune suppression is achieved through cross-mucosal induction of regulatory cytokines, regulatory DCs, macrophages, and CD4^+^ and CD8^+^ Tregs (76). In a recent study of murine *Trichuris muris* infection, Th2 cell proliferation was enhanced by early Treg depletion post-infection and by Treg depletion after establishment of infection (77). However, the ultimate effect of Tregs on pathogen persistence was clearly time dependent: both early and late Treg depletion enhanced Th2 responses and reduced Th1 responses, but while early Treg depletion resulted in enhanced clearance of infection, later, during infection, Treg depletion resulted in enhanced worm burden (77).

Importantly, in geohelminth-infected children *in vitro* depletion of CD4^+^Foxp3^+^CD25^HI^ T-cells increased not only antigen-specific proliferative responses but also IFNγ-production in response to *Plasmodium-*infected red blood cells (11). The *in vivo* effect of helminth coinfection on immunity against *Plasmodium* varies between studies, but helminth coinfection may be associated with protection against cerebral malaria, a state of severe hyper-inflammation (12). Latent tuberculosis infection (LTBI) individuals with hookworm (78) and filarial coinfection (79) had decreased Th1 and Th17 responses and increased Treg frequencies compared to parasite-uninfected LTBI individuals. Whether deworming has clinical impact on the course of TB disease is not clear: in TB patients with helminth coinfection, albendazole treatment decreased IL-10 levels, but there was no clinical improvement in TB after 2 months (80). Since (helminth-induced) Tregs are capable of exerting non-specific suppressive responses, research in malaria and TB (diseases where strong Th2 and Th1 responses are vital, respectively) will hopefully clarify the effect of Tregs across the boundaries of disease (Figure 1A), especially in settings where coinfection of helminths with malaria and/or TB is endemic.

## Tregs in Leprosy and Tuberculosis

### Tregs in leprosy, an ancient disease

Leprosy, caused by *M. leprae*, is an ancient, chronic, disabling, but curable disease affecting the skin, the peripheral nerves, the eyes, and mucosa of the respiratory tract (81). The clinical spectrum of the disease ranges from tuberculoid (TT) and borderline tuberculoid (BT) to borderline lepromatous (BL) and lepromatous leprosy (LL), where TT/BT is immunologically characterized by a strong Th1 response accompanied by limited growth of the bacillus (paucibacillary leprosy), whereas BL/LL is classically characterized by a predominant Treg/Th2 response, high antibody titers, absent granuloma formation, and thus poor containment of infection and clinical deterioration (82).

Though the exact mechanisms ruling this spectrum have not been elucidated, it is clear that Tregs play a part, and demonstration of the suppressive activity of CD4^+^ and CD8^+^ Tregs isolated from the skin and circulation of LL patients were among the first reports on “human T-suppressor cells” (6, 7). In the circulation of leprosy patients, both CD4^+^Foxp3^+^ and CD8^+^Foxp3^+^ T-cells were almost twofold increased compared to healthy contacts (83). Within the spectrum of disease, increased percentages of CD4^+^Foxp3^+^CD25^+^ and CD8^+^Foxp3^+^CD25^+^ T-cells have been demonstrated in the circulation of LL patients compared to BT patients or healthy contacts (82, 84). Also in lepromatous lesions, Foxp3^+^ T-cells were increasingly expressed in LL compared to TT/BT patients (82, 84). Suppression of the Th1 response by Tregs was demonstrated by enhanced *in vitro* IFNγ-production through depletion of CD25^+^ cells in a subset of LL patients (82). Both CD4^+^CD25^+^ derived IL-10 production and regulation through TGF-β have been described (85, 86).

A possible mechanism of Treg induction by *M. leprae*–infected DCs is the expression of the mycobacterial cell wall component PGL-1, that by association with the complement component C3 can steer toward Treg differentiation (87). Type 2 anti-inflammatory (CD163^+^) macrophages are important Treg inducers (17), possibly due to the action of ROS (88); indeed, a regulatory phenotype was described in monocytes stimulated with *M. leprae* (89). Recently, CD68^+^CD163^+^ cells were demonstrated in LL skin lesions with increased frequencies compared to BT/TT lesions (82). Intracellular pathways leading to enhanced Foxp3 expression in CD4^+^ T-cells have been described in association with progression of disease toward BL/LL, in addition to low Foxp3 ubiquitination (marked for intracellular degradation) (86). In T-cells isolated from LL patients, Foxp3 interacted with histone deacetylases and bound directly to the promotor regions of CD25 and CTLA-4 (90). The importance of this transcriptional regulation by Foxp3 within the immunological spectrum of disease is further supported by the fact that not only Treg frequencies are increased in LL compared to BT patients but also the intensity of expression (mean fluorescence intensity) of Foxp3 as determined by flow cytometry (83).

Thus, Tregs are clearly involved in the impairment of mycobacterial control. However, this does not necessarily equate to increased suppression of Th1 over Th2 responses toward the LL pole spectrum: gene expression profiling of PBMCs isolated from TT, LL, and borderline leprosy patients revealed decreased expression of both Th1 and Th2 genes in LL patients, but enhanced expression of CTLA-4 and TGFB1 (91). The authors further found overexpression of CBL-B, an E3 ubiquitin-ligase that after encounter with antigen is crucial in modulating T-cells toward activation vs. anergy, dependent on the presence or absence of co-stimulatory signals (92). Cbl-b, TGF-β, and CTLA-4 expression were molecularly related, as demonstrated by the dependency of Cbl-b expression on TGF-β and the decreased expression of Cbl-b after treatment with CTLA-4 siRNA (91). Within the paradigm of a generalized suppressed peripheral T-cell response associated with LL development, Tregs could thus play an important role in inducing and maintaining low cellular immune responsiveness (Figure 1A), although their impact on humoral (mostly but not exclusively Th2 related) responses remains less clear. Further work would be needed to clarify causal relationships, e.g., if Tregs are a cause or consequence of bacterial burden in LL disease (93).

### Tuberculosis: Early and late effects of Tregs

Pathogen-specific Tregs were induced by Mtb as demonstrated in a murine Mtb aerosol infection model, and these Tregs delayed priming of CD4^+^ and CD8^+^ T-cells in the pulmonary LNs, thereby delaying migration of these cells to the lung (94). Tregs were demonstrated in the lung, including in granulomas (95), and were shown to prevent pathogen clearance (96). Interestingly, in contrast to *Listeria monocytogenes*, pathogen-specific Treg expansion could be found in LNs only after Mtb infection (97). Thus, Mtb-induced Tregs contribute to the delayed onset of adaptive immunity that is observed in TB compared to other diseases and which allows establishment of infection (98, 99). The impact of Tregs on establishment of infection was further demonstrated in a murine study, where depletion of CD25^+^ cells early after Mtb infection – but not during chronic infection – decreased bacterial load and granuloma formation (100). However, it might also be that (pre-existing) Tregs have a beneficial role very early in infection, but also these data are only derived from animal experiments. In macaques, Tregs and IFNγ-producing effector T-cells expanded early after pulmonary TB infection, yet *in vivo* depletion of both IFNγ-producing and Tregs led to decreased resistance against granuloma progression (101). Analogous to the possibly beneficial role for Tregs in regulating LN in- and efflux during early murine HSV-infection (41, 42), it is conceivable that the presence of a very low level of (possibly pre-existing) Tregs before or in a very early state after Mtb infection might thus accommodate priming and subsequent emergence of a pro-inflammatory immune response. Clearly, further research will be needed to specify the impact of Tregs in various organs (102), early in (human) Mtb infection, and to differentiate their impact in early vs. chronic infection (Figure 1A).

Regulatory T-cells are also present in human Mtb infection as has been demonstrated extensively: Tregs could be isolated both from the circulation and from the site of infection in TB patients. In the circulation of TB patients, an increase in FOXP3 mRNA expression was found compared with healthy controls (103), and also an increase in CD4^+^ T-cell frequencies with regulatory phenotypes was demonstrated [defined as CD4^+^CD25^+/HI^ (103, 104), CD4^+^Foxp3^+^CD25^HI^ (105, 106), or CD4^+^CD25^HI^CD39^+^ (105)]. Tregs could be isolated from various Mtb-infected sites, including bronco-alveolar lavage (BAL) fluid, ascites, pericardial fluid, and pleural fluid; and FOXP3 mRNA expression levels and CD4^+^CD25^HI^ T-cell frequencies were increased stronger locally than systemically (in the circulation) (103, 107). In a study comparing TB cases with infected and uninfected TB contacts (defined by positive tuberculin-skin test (TST) and ELISPOT results), PBMCs from uninfected contacts had lower FOXP3 mRNA expression levels compared to TB cases, but higher FOXP3 expression levels compared to infected TB contacts; which according to the authors could signify migration of Tregs to the lungs during early infection, with a reappearance in the circulation during latent (established) infection (108). Also CD8^+^Foxp3^+^CD25^+^ Tregs were demonstrated in the circulation and BAL fluid of TB patients (107); and CD8^+^LAG-3^+^CCL4^+^ Tregs [lymphocyte activation gene-3 (LAG-3); CC chemokine ligand 4 (CCL4)] were shown by histological staining of infected LNs from TB patients (109). Furthermore, after stimulation with HLA-E restricted Mtb-derived peptides CD8^+^ Tregs could be isolated from PBMCs of *in vitro* mycobacterial purified protein derivative (PPD)-reactive donors (110, 111).

Elevated frequencies of circulating Tregs in TB patients declined during successful chemotherapy (106), in contrast, in patients with emerging MDR-TB circulating Treg frequencies remained persistently high (106). Other data on Tregs in MDR vs. normally resistant (NR)-TB are scarce and conflicting: similar frequencies of circulating CD4^+^Foxp3^+^ Tregs were found in MDR-TB patients compared to (NR-)TB patients (112); however, in another study comparing MDR-TB, NR-TB, and non-tuberculous mycobacteria (NTM) infections, increased *ex vivo* frequencies of Tregs were found in MDR-TB but also in NTM infections compared to NR-TB. This may reflect chronicity of infection in MDR-TB and NTM infection, which is often treated sub-optimally; however, the contrast reported by the authors between elevated serum IL-10 levels in MDR-TB patients vs. elevated serum TGF-β levels in NTM-infected patients could also suggest different subsets of Tregs or different suppressive effector pathways to be involved in MDR-TB vs. NTM (113).

### Tuberculosis: Tregs differentiate active from latent disease

CD4^+^Foxp3^+^CD25^+^ Tregs are increased in frequency in active TB compared to LTBI (107, 114), both in the circulation and in BAL fluid (107) (Figure 1A). A report on CD4^+^CD25^+^CD134^+^ T-cells in TB demonstrated differentiation between active and latent TB solely through the presence or absence of the CD39-molecule on this subset (115). Stasis of mycobacterial growth in macrophages, both monocyte-derived and alveolar, was suppressed by CD4^+^ Tregs (107). Depletion of CD4^+^Foxp3^+^CD25^HI^ T-cells increased IFNγ responses to the mycobacterial antigen heparin-binding hemagglutinin (HBHA) of patients with active TB *in vitro*, to the level observed in LTBI individuals (116). Treg frequency in the circulation of smear-positive TB patients was increased compared to smear-negative patients; however, this did not correlate with radiologic determination of extent of disease (112).

Pro-inflammatory signatures of CD8^+^ T-cells differentiated between latent infection and active TB disease (117), and also *in vitro* an association was found between burden of infection of cells and lysis by cytotoxic CD8^+^ T-cells (118). The frequency of CD8^+^ T-cells producing IL-10 or TGF-β was increased in active TB patients compared to latently infected or control subjects (119). In this study, CD8^+^, CD8^+^IFN-γ^+^, and CD8^+^IL-17^+^ T-cell numbers were similar between groups, and were – interestingly – not dependent on sputum bacillary load, while sputum bacillary load was positively associated with specific regulatory cytokine expression in CD8^+^ T-cells, and negatively associated with CD8^+^ granzyme B expression (119). However, in another study, the frequency of CD8^+^Foxp3^+^CD25^+^ Tregs did not differ between active vs. latent TB, or between cells isolated from the circulation vs. cells isolated from BAL fluid (107). The differences between these reports may be explicated by differences in regulatory markers that were studied, or by methods that were used: in the former study, cells were stimulated with Mtb specific antigen for 96 h, while in the latter study, cells were PPD-stimulated for 12 h. CD8^+^ Tregs are relatively understudied compared to CD4^+^ Tregs in mycobacterial infection (5), and this clearly points to the need for more (uniform) research into these possibly important regulators and/or markers of activity of disease. Of note, CD8^+^ Tregs were found at the disease site in mice, and progression of disease correlated with accumulation of IL-10-secreting CD8^+^ T-cells in granulomas (120).

Instead of being a steady state of infection, latent TB comprises a dynamic spectrum with supposedly increasing rates of subclinical Mtb replication and inflammation extending eventually to active TB. Serial IGRA testing has been proposed as an indicator of human host resistance in latent TB. Using serial testing, a consistently negative test in TB-exposed individuals would likely indicate strong resistance to infection, a consistently positive test (recent) active infection, and (repeated) test conversions (positive to negative, possibly followed by conversion, etc.) changing dynamics of infection and control of bacterial load. In a comparison of T-cell subsets between IGRA-consistently positive and consistently negative TB-case contacts, CD4^+^Foxp3^+^ and CD4^+^CTLA-4^+^ Tregs were increased in TB-case contacts with consistently positive IGRA-tests, possibly indicating Treg interference with host resistance in the development of active infection (121).

### Tuberculosis: Tregs in extra-pulmonary disease

A minority of TB cases present with extra-pulmonary disease or extra-pulmonary involvement following pulmonary infection, and it is assumed that this represents failure of the immune system to contain infection (122). Multiple studies indicate involvement of Tregs in dissemination of disease (Figure 1A). An increase in FOXP3 mRNA expression has been described in PBMCs from patients with extra-pulmonary TB (disseminated and lymphatic TB) compared to pulmonary TB (103). In a comparison of TB pleural effusion and miliary TB, representing in this case containment vs. dissemination of disease, elevated FOXP3 mRNA expression levels and frequencies of CD4^+^Foxp3^+^CD25^+^ T-cells were found in cells isolated from miliary disease sites (123). Another study confirmed an increase in CD4^+^ Treg frequencies in patients previously treated for extra-pulmonary TB compared to pulmonary TB, but reported an analogous increase in CD4^+^ activation markers (124). In TB pleurisy, CD4^+^Foxp3^+^CD25^HI^ Treg frequencies were increased in pleural fluid compared to the circulation (125, 126), and Tregs suppressed IFNγ-expression in CD4^+^ and CD8^+^ T-cells (126). Pleural CD39^+^ Tregs inhibited generation of Th17 cells, which could be reversed *in vitro* by antagonizing TGF-β through the addition of latency-associated peptide (LAP) (127). Mtb infection of the pleurae favored Treg migration into the pleural exudate when compared to other causes of pleurisy: tuberculous pleural fluid, but not effusions from other bacterial origin, or transudates, had high concentrations of the chemoattractant CCL22, which is chemotactic for Treg migration *in vitro*, and an increase in CD4^+^CD25^HI^ T-cell frequency compared to the circulation (125). Intercellular adhesion molecule-1 (ICAM-1) and vascular cell adhesion molecule-1 (VCAM-1) on pleural mesothelial cells regulated migration of leukocytes from the circulation into the pleural fluid; however, these molecules also seemed to favor (non-antigen-specific) expansion of Tregs (128).

In TB lymphadenitis in children, CD4^+^Foxp3^+^ T-cells were demonstrated in the LNs, and quantitative mRNA analysis demonstrated induction of TGFβ and IL13, but not of IFNγ, TNFα, or IL-17 (129). Data on frequency and function of Tregs in other forms of TB disease, such as bone TB, urogenital TB, or TB of the central nervous system (CNS) are scarce. It is, however, conceivable that the interplay of Tregs and Mtb may differ in infections at immune-privileged sites, such as the CNS or the eye. The assessment of anti-inflammatory mechanisms could be highly relevant in regard to CNS-immune reconstitution syndromes, given their often disastrous outcomes (130). Several studies have associated plasma biomarkers and CD4^+^ T-cell activation with the development of HIV-associated immune reconstitution inflammatory syndromes (IRIS), but did not find an association with (CD4^+^) Treg frequencies, both in the development of cryptococcal-IRIS disease (131) and TB-IRIS disease (131, 132). TB-IRIS may either be “unmasking” (of an occult infection) or “paradoxical” (worsening of a known infection during retroviral treatment) hyper-inflammation: decreased serum IL-10 levels were found in paradoxical compared to unmasking syndromes (133). Interestingly, this might represent Treg function, not Treg phenotype: a study in patients developing symptoms of *Mycobacterium avium* and *intracellulare* complex-infection, following commencement of retroviral treatment, reported a significant expansion of CD4^+^Foxp3^+^CD25^+^CD127^LO^ Tregs, but reduced functional capacity and diminished IL-10 secretion of these cells in *in vitro* suppression assays (134).

### Tuberculosis: Tregs in the clinic

Tregs may interfere with clinical diagnosis of TB (Figure 1A). Classically, diagnosing TB has relied for decades on the TST, testing cell-mediated immunity against intradermally injected Mtb-derived tuberculin PPD. Skin anergy is defined as the absence of dermal reactivity in otherwise confirmed Mtb infection. *In vitro* PPD stimulation of cells isolated from PPD-reactive TB patients induced both IL-10 and IFNγ-production; however, cells from anergic TB patients produced only IL-10 but not IFNγ (135). Reduced levels of IFNγ and IL-2, and increased levels of IL-10 in anergic compared to PPD-reactive TB patients were confirmed in another study. This anergy was found only after *in vitro* stimulation with PPD – but not unrelated antigens, indicating an antigen-specific anergic reaction (136). Suppression of IL-2- and TNFα-production was accompanied by CD8^+^ T-cell expansion and high levels of IL-10 in anergic TB patients, and CD8^+^ T-cell depletion and blocking of IL-10 reversed this suppression (137).

A direct effect of Treg-mediated suppression on interferon-γ release assays (IGRAs), such as the in-tube QuantiFERON Test, has so far not been established. Nevertheless, several studies have described “rescue” of mycobacterial-specific IFNγ production by Treg depletion in Mtb-infected individuals (104, 105, 114, 138). Interestingly, depletion of CD25^+^ T-cells increased IFNγ production by PBMCs in Mtb-infected individuals, but did not increase the production of IL-17A (114). Yet, pleural CD39^+^ Tregs (CD4^+^CD25^+^CD39^+^CD127^−^) inhibited Th17 differentiation (127), and an inverse correlation between production of IL-17A and CD39-expressing Tregs has been described after vaccination (139, 140). CD39 expression on Tregs may thus be more closely linked to suppression of IL-17 production compared to cells expressing CD25, but this needs further clarification. Also the extent of TB infection as determined by chest X-ray (CXR) scoring was associated with T-cell modulation: in a study dividing patients by severity of disease by CXR, double-negative (DN, CD4^−^CD8^−^) TCRγδ T-cells from patients with severe disease displayed a modulatory profile with high IL-10 production, in contrast to patients with less severe disease, where TCRγδ DN T-cells displayed a pro-inflammatory cytokine profile with high IFNγ (141).

During TB therapy, circulating CD4^+^ Treg frequencies declined as mentioned; however, this was only noted following chemotherapy for pulmonary TB (Figure 1A) (106, 142, 143). In contrast, an increase was noted during extra-pulmonary TB treatment (143, 144). Differences between forms of disease possibly represent differences in compartmentalization of Tregs, or heterogeneous kinetics of Treg contraction following decrease of bacterial burden. TB patients in which MDR-TB emerged during therapy had persistent circulating Treg frequencies (106), which could be analogous to a phenomenon observed during IFNα therapy for chronic HBV infection: therapy non-responders were characterized by an increase in CD4^+^CD25^+^ T-cells and IL-10-producing cells (145). Thus, circulating Treg frequencies might be used as parameter of therapy response in specific states of TB disease.

## Tregs in Vaccination Against Tuberculosis

Even in early life, immunoregulatory mechanisms, including Tregs, may dampen vaccine-induced immunity (146). We describe here how immunogenicity of TB vaccines may be influenced by Tregs, induced by the vaccination itself, by closely related pathogens, or induced by unrelated pathogens (Figure 1B). *M. bovis* BCG, the only available vaccine against TB, is a live bacterial vaccine aimed at inducing effective T-cell responses, yet BCG itself also induces Tregs (5). This ability to induce Tregs could limit its ability to induce optimal protective immunity against TB; it is, however, conceivable that future medicine may be able to tailor BCG-induced Tregs to regulate hyperinflammation.

### Tregs induced by vaccination: *M. bovis Bacillus* Calmette–Guérin

Bacillus Calmette–Guérin, the only licensed vaccine against TB since 1921, was derived from virulent *M. bovis* by years of continuous *in vitro* passage. Estimates are that BCG has been given >3 billion times since its introduction, and it is part of the WHO Expanded Program for Immunization (EPI). BCG was used in one of the first experiments establishing the idea of “suppressor cells” interfering with control of infection: transfer of thymocytes from BCG-immunized rats suppressed immune responses in naïve recipient rats against new BCG infection (147). Though BCG-vaccination induces CD4^+^ and CD8^+^ effector T-cell responses in newborns (148, 149) and protects them from disseminated forms of disease, it does not induce consistent protection against pulmonary TB, especially in adults (150). We have previously hypothesized that one explanation for this lack of protection is the induction of Tregs by the vaccine among various other hypotheses (5). In a large cohort of 5675 South-African infants who had been vaccinated at birth, stimulation of whole blood with mycobacterial antigens at 10 weeks of age resulted in production of IFNγ or IL-10, but not both (151). CD4^+^CD25^+^ Treg cells were demonstrated in another study in BCG-vaccinated infants, and depletion of these Treg cells resulted in lower IL-10 levels in PPD-stimulated cell cultures (152). IL-10-producing CD4^+^ T-cells have been demonstrated in previously BCG-vaccinated adult donors, and *in vitro* suppression of target cell proliferation could be reversed by a blocking αIL-10-antibody (153).

CD8^+^ Tregs are generally less studied compared to CD4^+^ Tregs, especially in infectious diseases (5). We have previously studied the presence, phenotype, and suppressive activity of CD8^+^ Treg cells among live BCG-stimulated PBMCs of *in vitro* PPD-responsive donors. Surprisingly, we found a significantly higher expression of regulatory markers on live (but not killed) BCG-activated CD8^+^ T-cells compared to CD4^+^ T-cells, and there was significant enrichment of CD8^+^ Treg cells within the BCG-activated CD25^+^ T-cell compartment (154). Also, suppressive activity was dominantly present in live BCG-activated CD8^+^, but not in live BCG-activated CD4^+^ T-cells (154). CD8^+^ Treg cells isolated from live BCG-stimulated PBMCs were enriched for expression of LAG-3 and CCL4, co-expressed CD25 and Foxp3, and inhibited Th1 cell proliferation (109). Inhibition was partly mediated by secretion of CCL4, which reduced Ca^2+^-influx early after TCR triggering (109). We have additionally described expression of CD39 on live BCG-activated CD8^+^ Treg cells, and a direct involvement of CD39 in mediating suppression by CD8^+^ Tregs, as both the chemical CD39 antagonist ARL 67156 and a blocking αCD39-antibody were able to partly inhibit the suppressive activity of CD8^+^CD39^+^ Tregs (155). Of note, CD8^+^ Tregs could only be demonstrated in donors primed *in vivo* with mycobacteria, indicating a memory recall response following *in vitro* BCG stimulation. Taken together, our work identified at least two different mechanisms by which BCG-activated CD8^+^ Tregs could inhibit Th1 responses, via CCL4 and via CD39. Despite the above findings and despite the fact that CD8 was originally identified as a marker of Treg cells, then coined T-suppressor cells, pathogen-activated CD8^+^ Tregs still remain significantly understudied compared to CD4^+^ Tregs. It is important to note here that *in vitro* stimulation with live BCG preferentially activated CD8^+^ Tregs (154), while stimulation with killed BCG (or PPD) seems to activate different populations.

### Tregs induced by new TB-candidate vaccines

Regulatory T-cell induction has been demonstrated in several TB-vaccine candidate trials. After M72/AS01-vaccination of South-African healthy adults, Tregs expanded concurrently with cytokine-producing pro-inflammatory CD4^+^ T-cells (156). Circulating CD4^+^CD25^+^Foxp3^+^ T-cells were demonstrated after vaccination with another TB-vaccine candidate, modified vaccinia Ankara-85A (MVA85A). Interestingly, CD4^+^CD25^+^Foxp3^+^ T-cells were increased in recipients with low antigen 85A-specific IFNγ-responses compared to high IFNγ-responders (157). Also, the frequency of CD4^+^CD25^+^CD39^+^ T-cells was inversely related to IL-17A production *in vitro* (139). IL2RA mRNA expression on the day of vaccination and CTLA-4 expression 2 days after vaccination inversely correlated with the magnitude of the IFNγ ELISPOT response induced by MVA85A vaccination in healthy British adults, pointing to a possible role for Tregs very early or even before vaccination (157). In African infants vaccinated with MVA85A, an early and strong innate response was associated with enhanced IFNγ ELISPOT responses; thus, the authors concluded that Treg modulation of vaccine responses could differ between populations, and that more research is needed to explain these differences and the impact on vaccine efficacy (158). Assessment should, however, include possible dissimilarities between long-term effects of Tregs and early after vaccination.

### Other Tregs can modulate TB-vaccine-induced responses

Regulatory T-cells induced by other microbes can likely alter immunogenicity of TB vaccines. Exposure to environmental mycobacteria may decrease TB-vaccine efficacy through cross-reaction of antigens (94). Pre-existing immune responses can either “block” or “mask” the BCG-induced immune response, possibly explaining the decreased vaccine efficacy of BCG in developing countries, where there is a higher prevalence of environmental mycobacteria (159). Another potential explanation for decreased vaccine efficacy is induction of Tregs by environmental mycobacteria (160). Priming mice with *M. chelonae* before BCG-vaccination increased Foxp3 expression on BCG-specific CD4^+^CD25^+^ T-cells compared to non-sensitized mice, and CD4^+^CD25^+^ T-cells of sensitized mice decreased immune responses *in vitro* (161). Adoptive transfer of CD4^+^CD25^+^ T-cells into naïve mice suppressed IL-2-production in the lungs, and enhanced IL-10 after BCG-vaccination (161). Suppression after murine sensitization was reversed by a blocking αCD25-mAb during challenge, indicating active involvement of cross-reactive Tregs during vaccination (162).

Modulation of DC TLRs by helminth molecules lead to increased Th2 and Treg responses, which possibly decreases vaccine efficacy in developing countries, where also the majority of the one billion helminth-infected people live (12). Tregs induced by helminths in mucosa-associated lymphoid tissue (MALT) may migrate to other sites, exerting non-specific suppressive effects and preventing clearance of Mtb at distant sites as well (163). Although the frequency of CD4^+^Foxp3^+^CD25^HI^ T-cells was similar in helminth-infected and non-infected Indonesian children, BCG-specific (and as mentioned, also *Plasmodium falciparum*-specific) proliferative responses were increased after depletion of CD4^+^CD25^HI^ T-cells in helminth-infected children only, pointing to differences in suppressive capacity induced by helminth infection (11). Deworming increased BCG immunogenicity *in vivo* and was accompanied by changes in TGF-β, but notably not by changes in Th2 cytokines (164).

### Modulating the modulators: Future prospects for Tregs in TB-vaccination

The ability of BCG to induce Tregs may in the future be exploited to benefit the human host in the contexts of auto-immune and/or hyper-inflammation-related diseases. This has been noted in a murine model of Parkinson’s disease, where protection against nerve damage was induced by BCG-vaccination through Tregs (165). Also in experimental auto-immune encephalomyelitis, myelin oligodendrocyte glycoprotein-specific IFNγ-producing CD4^+^ T-cells, and both specific and non-specific CD4^+^IL-17^+^ T-cells in the CNS, were suppressed by cerebral BCG infection (166). Other murine studies have demonstrated BCG-induced suppression of asthma responses and dampening of colitis (167, 168). Further research will hopefully elucidate if and how these findings can be translated to the human situation.

Interestingly, mucosal vaccination of macaques with a vaccine consisting of inactivated simian immunodeficiency virus (SIV) and a live bacterial adjuvant (BCG or *Lactobacillus*) generated HLA-E restricted, non-cytolytic CD8^+^ Tregs (169). After challenge with SIV infection, these CD8^+^ Tregs suppressed proliferation of infected CD4^+^ T-cells, thereby protecting almost all vaccinated macaques for up to 4 years after vaccination (169). As mentioned, in acute viral infection, Tregs could have a beneficial role to play, such as in acute SIV/HIV infection where Tregs decrease proliferation of infected cells at mucosal surfaces (44).

In a TB-vaccination context, however, it may be crucial to avoid excessive Treg induction by the vaccine. Analogous to the reduced burden of TB observed in mice following treatment with chemical compounds inhibiting Treg and Th2 induction prior to infection (170), a similar approach was tested in murine BCG-vaccination: chemical inhibition of Treg induction increased BCG-mediated protection against pulmonary TB in mice and favored central-memory T-cell induction (long-lived vaccine responses) (171). Blocking the IL-10-receptor with an αIL-10-receptor antibody increased BCG-induced Th1, Th17, innate lymphoid IFNγ, and IL-17 responses in mice, leading to enhanced protection against TB (172). An additional, important role for IL-22 producing NK cells through lysing of CD4^+^ Tregs was described, and addition of IL-22 also increased Th1 vaccine-induced responses (173). In contrast, only moderate efficacy of treatment with a blocking αCD25-antibody on BCG-vaccine efficacy was described (174). It is possible that blocking CD25 results in partial Treg depletion while other Treg subsets could survive during such treatment. However, CD25 is expressed also by activated T-helper cells such that CD25-depletion may additionally also deplete essential effector cells of protective immunity. Regardless, even after selective deletion of all Foxp3^+^ cells, homeostatic expansion may occur from a small subset of remaining Tregs (175). Since various Treg marker-expressing subsets exist, this points to the importance of assessing the dynamics and fluidity of various subsets within the Treg compartment, in order to improve vaccine design by effective modulation of Treg activity and function. Compounds inhibiting Treg induction or blocking “upstream” signaling through the IL-10-receptor could improve vaccine efficacy. Other options would include the addition of adjuvant antagonists of chemokine receptors expressed by Tregs, as described for a CCR4 antagonist that blocked CD4^+^ Tregs and increased *in vitro* responses to MVA85A and recombinant HBV surface antigen vaccination (176), or the inclusion of TLR-agonists combined with agents selectively blocking TLR-induced anti-inflammatory signaling pathways in DCs (177). Future studies may integrate these findings to increase TB-vaccine-induced protective immunity through manipulation of the manipulators, and hopefully translate these findings ultimately to the human situation.

## Concluding Remarks and Future Directions

For many pathogens, induction, expansion, recruitment, or inhibition of Tregs has been demonstrated. *Mycobacterium leprae* and *Mycobacterium tuberculosis* are master manipulators of human immunity and are able to establish chronic infection among others by activating immune regulation. The effects of Tregs impact on clinical symptoms and performance of immunodiagnostic assays, differ in acute vs. chronic diseases and can suppress protective immunity and vaccine immunogenicity. Importantly, this can partly be the result of cross-suppression from Tregs induced by unrelated pathogens, possibly even by non-pathogenic microbes. This is particularly important in endemic settings, e.g., settings endemic for both helminths, TB, malaria, and HIV.

Through precisely (and timely) targeted Treg manipulation, vaccine-induced protective immunity may be enhanced. Most data are necessarily derived from murine studies, and need to be translated to the human situation. This should also offer opportunities for new immunotherapeutic vaccines for the treatment of inflammatory disorders, e.g., auto-immune diseases, and for the design of vaccines aimed at interfering with acute (viral) infection. Through manipulating the manipulators, increased immunity against infectious diseases may be achieved.

## Author Contributions

All authors fulfill the criteria for authorship.

## Conflict of Interest Statement

The authors declare that the research was conducted in the absence of any commercial or financial relationships that could be construed as a potential conflict of interest.

## References

[1] Belkaid Y Tarbell K. Regulatory T cells in the control of host-microorganism interactions. Annu Rev Immunol (2009) 27:551–89. 10.1146/annurev.immunol.021908.132723 19302048

[2] Sutmuller RP Morgan ME Netea MG Grauer O Adema GJ. Toll-like receptors on regulatory T cells: expanding immune regulation. Trends Immunol (2006) 27:387–93. 10.1016/j.it.2006.06.005 16814607

[3] Maizels RM Smith KA Regulatory T cells in infection. Adv Immunol (2011) 112:73–136 10.1016/B978-0-12-387827-4.00003-6 22118407 PMC7150045

[4] Abbas AK Benoist C Bluestone JA Campbell DJ Ghosh S Hori S Regulatory T cells: recommendations to simplify the nomenclature. Nat Immunol (2013) 14:307–8 10.1038/ni.2554 23507634

[5] Joosten SA Ottenhoff TH. Human CD4 and CD8 regulatory T cells in infectious diseases and vaccination. Hum Immunol (2008) 69:760–70. 10.1016/j.humimm.2008.07.017 18835413

[6] Ottenhoff TH Elferink DG Klatser PR de Vries RR. Cloned suppressor T cells from a lepromatous leprosy patient suppress *Mycobacterium leprae* reactive helper T cells. Nature (1986) 322:462–4. 10.1038/322462a0 2426597

[7] Modlin RL Kato H Mehra V Nelson EE Fan XD Rea TH Genetically restricted suppressor T-cell clones derived from lepromatous leprosy lesions. Nature (1986) 322:459–61. 10.1038/322459a0 2942780

[8] Kapp JA Bucy RP. CD8+ suppressor T cells resurrected. Hum Immunol (2008) 69:715–20. 10.1016/j.humimm.2008.07.018 18817830

[9] Wing JB Sakaguchi S. Multiple treg suppressive modules and their adaptability. Front Immunol (2012) 3:178. 10.3389/fimmu.2012.00178 22754556 PMC3386489

[10] Vignali DA Collison LW Workman CJ How regulatory T cells work. Nat Rev Immunol (2008) 8:523–32 10.1038/nri2343 18566595 PMC2665249

[11] Wammes LJ Hamid F Wiria AE de Gier B Sartono E Maizels RM Regulatory T cells in human geohelminth infection suppress immune responses to BCG and *Plasmodium falciparum*. Eur J Immunol (2010) 40:437–42. 10.1002/eji.200939699 20063313

[12] van Riet E Hartgers FC Yazdanbakhsh M. Chronic helminth infections induce immunomodulation: consequences and mechanisms. Immunobiology (2007) 212:475–90. 10.1016/j.imbio.2007.03.009 17544832

[13] Qureshi OS Zheng Y Nakamura K Attridge K Manzotti C Schmidt EM Trans-endocytosis of CD80 and CD86: a molecular basis for the cell-extrinsic function of CTLA-4. Science (2011) 332:600–3. 10.1126/science.1202947 21474713 PMC3198051

[14] Dwyer KM Deaglio S Gao W Friedman D Strom TB Robson SC. CD39 and control of cellular immune responses. Purinergic Signal (2007) 3:171–80. 10.1007/s11302-006-9050-y 18404431 PMC2096766

[15] Murray PJ Wynn TA. Protective and pathogenic functions of macrophage subsets. Nat Rev Immunol (2011) 11:723–37. 10.1038/nri3073 21997792 PMC3422549

[16] Verreck FA de Boer T Langenberg DM Hoeve MA Kramer M Vaisberg E Human IL-23-producing type 1 macrophages promote but IL-10-producing type 2 macrophages subvert immunity to (myco)bacteria. Proc Natl Acad Sci U S A (2004) 101:4560–5. 10.1073/pnas.0400983101 15070757 PMC384786

[17] Savage ND de Boer T Walburg KV Joosten SA van MK Geluk A Human anti-inflammatory macrophages induce Foxp3+ GITR+ CD25+ regulatory T cells, which suppress via membrane-bound TGFbeta-1. J Immunol (2008) 181:2220–6. 10.4049/jimmunol.181.3.2220 18641362

[18] Gupta N Hegde P Lecerf M Nain M Kaur M Kalia M Japanese encephalitis virus expands regulatory T cells by increasing the expression of PD-L1 on dendritic cells. Eur J Immunol (2014) 44:1363–74. 10.1002/eji.201343701 24643627

[19] Trinath J Maddur MS Kaveri SV Balaji KN Bayry J *Mycobacterium tuberculosis* promotes regulatory T-cell expansion via induction of programmed death-1 ligand 1 (PD-L1, CD274) on dendritic cells. J Infect Dis (2012) 205:694–6 10.1093/infdis/jir820 22238465

[20] Periasamy S Dhiman R Barnes PF Paidipally P Tvinnereim A Bandaru A Programmed death 1 and cytokine inducible SH2-containing protein dependent expansion of regulatory T cells upon stimulation with *Mycobacterium tuberculosis*. J Infect Dis (2011) 203:1256–63. 10.1093/infdis/jir011 21383382 PMC3069733

[21] Siddiqui KF Amir M Gurram RK Khan N Arora A Rajagopal K Latency-associated protein Acr1 impairs dendritic cell maturation and functionality: a possible mechanism of immune evasion by *Mycobacterium tuberculosis*. J Infect Dis (2014) 209:1436–45. 10.1093/infdis/jit595 24218502

[22] Dubois-Colas N Petit-Jentreau L Barreiro LB Durand S Soubigou G Lecointe C Extracellular adenosine triphosphate affects the response of human macrophages infected with *Mycobacterium tuberculosis*. J Infect Dis (2014) 210:824–33. 10.1093/infdis/jiu135 24604822

[23] Ohta A Sitkovsky M. Role of G-protein-coupled adenosine receptors in downregulation of inflammation and protection from tissue damage. Nature (2001) 414:916–20. 10.1038/414916a 11780065

[24] Hall CH Kassel R Tacke RS Hahn YS. HCV+ hepatocytes induce human regulatory CD4+ T cells through the production of TGF-beta. PLoS One (2010) 5:e12154. 10.1371/journal.pone.0012154 20730048 PMC2921368

[25] Beswick EJ Pinchuk IV Earley RB Schmitt DA Reyes VE. Role of gastric epithelial cell-derived transforming growth factor beta in reduced CD4+ T cell proliferation and development of regulatory T cells during *Helicobacter pylori* infection. Infect Immun (2011) 79:2737–45. 10.1128/IAI.01146-10 21482686 PMC3191950

[26] Mertens J Fabri M Zingarelli A Kubacki T Meemboor S Groneck L *Streptococcus pneumoniae* serotype 1 capsular polysaccharide induces CD8CD28 regulatory T lymphocytes by TCR crosslinking. PLoS Pathog (2009) 5:e1000596. 10.1371/journal.ppat.1000596 19779562 PMC2742891

[27] Grainger JR Smith KA Hewitson JP McSorley HJ Harcus Y Filbey KJ Helminth secretions induce de novo T cell Foxp3 expression and regulatory function through the TGF-beta pathway. J Exp Med (2010) 207:2331–41. 10.1084/jem.20101074 20876311 PMC2964568

[28] Sharma S Rajasagi NK Veiga-Parga T Rouse BT. Herpes virus entry mediator (HVEM) modulates proliferation and activation of regulatory T cells following HSV-1 infection. Microbes Infect (2014) 16:648–60. 10.1016/j.micinf.2014.06.005 24956596 PMC4150749

[29] Ji J Cloyd MW. HIV-1 binding to CD4 on CD4+CD25+ regulatory T cells enhances their suppressive function and induces them to home to, and accumulate in, peripheral and mucosal lymphoid tissues: an additional mechanism of immunosuppression. Int Immunol (2009) 21:283–94. 10.1093/intimm/dxn146 19208751 PMC2645780

[30] Harding CV Boom WH. Regulation of antigen presentation by *Mycobacterium tuberculosis*: a role for Toll-like receptors. Nat Rev Microbiol (2010) 8:296–307. 10.1038/nrmicro2321 20234378 PMC3037727

[31] Saraav I Singh S Sharma S. Outcome of *Mycobacterium tuberculosis* and toll-like receptor interaction: immune response or immune evasion? Immunol Cell Biol (2014) 92:741–6. 10.1038/icb.2014.52 24983458

[32] McBride A Konowich J Salgame P. Host defense and recruitment of Foxp3(+) T regulatory cells to the lungs in chronic *Mycobacterium tuberculosis* infection requires toll-like receptor 2. PLoS Pathog (2013) 9:e1003397. 10.1371/journal.ppat.1003397 23785280 PMC3681744

[33] Hisaeda H Tetsutani K Imai T Moriya C Tu L Hamano S Malaria parasites require TLR9 signaling for immune evasion by activating regulatory T cells. J Immunol (2008) 180:2496–503. 10.4049/jimmunol.180.4.2496 18250459

[34] O’Neill LA Hardie DG. Metabolism of inflammation limited by AMPK and pseudo-starvation. Nature (2013) 493:346–55. 10.1038/nature11862 23325217

[35] Schiering C Krausgruber T Chomka A Frohlich A Adelmann K Wohlfert EA The alarmin IL-33 promotes regulatory T-cell function in the intestine. Nature (2014) 513:564–8. 10.1038/nature13577 25043027 PMC4339042

[36] Arpaia N Campbell C Fan X Dikiy S van der V deRoos P Metabolites produced by commensal bacteria promote peripheral regulatory T-cell generation. Nature (2013) 504:451–5. 10.1038/nature12726 24226773 PMC3869884

[37] Furusawa Y Obata Y Fukuda S Endo TA Nakato G Takahashi D Commensal microbe-derived butyrate induces the differentiation of colonic regulatory T cells. Nature (2013) 504:446–50. 10.1038/nature12721 24226770

[38] Veiga-Parga T Sehrawat S Rouse BT. Role of regulatory T cells during virus infection. Immunol Rev (2013) 255:182–96. 10.1111/imr.12085 23947355 PMC3748387

[39] Manangeeswaran M Jacques J Tami C Konduru K Amharref N Perrella O Binding of hepatitis A virus to its cellular receptor 1 inhibits T-regulatory cell functions in humans. Gastroenterology (2012) 142:1516–25. 10.1053/j.gastro.2012.02.039 22430395 PMC3367104

[40] Luhn K Simmons CP Moran E Dung NT Chau TN Quyen NT Increased frequencies of CD4+ CD25(high) regulatory T cells in acute dengue infection. J Exp Med (2007) 204:979–85. 10.1084/jem.20061381 17452519 PMC2118571

[41] Lund JM Hsing L Pham TT Rudensky AY. Coordination of early protective immunity to viral infection by regulatory T cells. Science (2008) 320:1220–4. 10.1126/science.1155209 18436744 PMC2519146

[42] Kassiotis G O’Garra A Immunology. Immunity benefits from a little suppression. Science (2008) 320:1168–9 10.1126/science.1159090 18511677

[43] Moreno-Fernandez ME Rueda CM Rusie LK Chougnet CA. Regulatory T cells control HIV replication in activated T cells through a cAMP-dependent mechanism. Blood (2011) 117:5372–80. 10.1182/blood-2010-12-323162 21436067 PMC3109711

[44] Haase AT. Perils at mucosal front lines for HIV and SIV and their hosts. Nat Rev Immunol (2005) 5:783–92. 10.1038/nri1706 16200081

[45] Graham JB Da CA Lund JM. Regulatory T cells shape the resident memory T cell response to virus infection in the tissues. J Immunol (2014) 192:683–90. 10.4049/jimmunol.1202153 24337378 PMC3894741

[46] Aalaei-Andabili SH Alavian SM. Regulatory T cells are the most important determinant factor of hepatitis B infection prognosis: a systematic review and meta-analysis. Vaccine (2012) 30:5595–602. 10.1016/j.vaccine.2012.06.063 22781305

[47] Losikoff PT Self AA Gregory SH. Dendritic cells, regulatory T cells and the pathogenesis of chronic hepatitis C. Virulence (2012) 3:610–20. 10.4161/viru.21823 23076334 PMC3545943

[48] Self AA Losikoff PT Gregory SH. Divergent contributions of regulatory T cells to the pathogenesis of chronic hepatitis C. Hum Vaccin Immunother (2013) 9:1569–76. 10.4161/hv.24726 23732899 PMC3974886

[49] Riezu-Boj JI Larrea E Aldabe R Guembe L Casares N Galeano E Hepatitis C virus induces the expression of CCL17 and CCL22 chemokines that attract regulatory T cells to the site of infection. J Hepatol (2011) 54:422–31. 10.1016/j.jhep.2010.07.014 21129807

[50] Chevalier MF Weiss L. The split personality of regulatory T cells in HIV infection. Blood (2013) 121:29–37. 10.1182/blood-2012-07-409755 23043072

[51] Moreno-Fernandez ME Joedicke JJ Chougnet CA. Regulatory T cells diminish HIV infection in dendritic cells – conventional CD4(+) T cell clusters. Front Immunol (2014) 5:199. 10.3389/fimmu.2014.00199 24847325 PMC4021135

[52] Nikolova M Carriere M Jenabian MA Limou S Younas M Kok A CD39/adenosine pathway is involved in AIDS progression. PLoS Pathog (2011) 7:e1002110. 10.1371/journal.ppat.1002110 21750674 PMC3131268

[53] Schulze Zur WJ Thomssen A Hartjen P Toth I Lehmann C Meyer-Olson D Comprehensive analysis of frequency and phenotype of T regulatory cells in HIV infection: CD39 expression of FoxP3+ T regulatory cells correlates with progressive disease. J Virol (2011) 85:1287–97. 10.1128/JVI.01758-10 21047964 PMC3020516

[54] Johanns TM Ertelt JM Rowe JH Way SS. Regulatory T cell suppressive potency dictates the balance between bacterial proliferation and clearance during persistent *Salmonella* infection. PLoS Pathog (2010) 6:e1001043. 10.1371/journal.ppat.1001043 20714351 PMC2920851

[55] Monack DM. *Helicobacter* and *Salmonella* persistent infection strategies. Cold Spring Harb Perspect Med (2013) 3:a010348. 10.1101/cshperspect.a010348 24296347 PMC3839601

[56] Neill DR Coward WR Gritzfeld JF Richards L Garcia-Garcia FJ Dotor J Density and duration of pneumococcal carriage is maintained by transforming growth factor beta1 and T regulatory cells. Am J Respir Crit Care Med (2014) 189:1250–9. 10.1164/rccm.201401-0128OC 24749506 PMC4225851

[57] Cook KW Letley DP Ingram RJ Staples E Skjoldmose H Atherton JC CCL20/CCR6-mediated migration of regulatory T cells to the *Helicobacter pylori*-infected human gastric mucosa. Gut (2014) 63:1550–9. 10.1136/gutjnl-2013-306253 24436142 PMC4173663

[58] Lina TT Alzahrani S Gonzalez J Pinchuk IV Beswick EJ Reyes VE. Immune evasion strategies used by *Helicobacter pylori*. World J Gastroenterol (2014) 20:12753–66. 10.3748/wjg.v20.i36.12753 25278676 PMC4177461

[59] Round JL Mazmanian SK. Inducible Foxp3+ regulatory T-cell development by a commensal bacterium of the intestinal microbiota. Proc Natl Acad Sci U S A (2010) 107:12204–9. 10.1073/pnas.0909122107 20566854 PMC2901479

[60] Santos RC Gomes-Santos AC Garcias MT de AM Diniz LT Mariadassou M Local and systemic immune mechanisms underlying the anti-colitis effects of the dairy bacterium *Lactobacillus delbrueckii*. PLoS One (2014) 9:e85923. 10.1371/journal.pone.0085923 24465791 PMC3897545

[61] Nakamizo S Egawa G Honda T Nakajima S Belkaid Y Kabashima K Commensal bacteria and cutaneous immunity. Semin Immunopathol (2014) 37:73–80 10.1007/s00281-014-0452-6 25326105

[62] Marsland BJ Gollwitzer ES Host-microorganism interactions in lung diseases. Nat Rev Immunol (2014) 14:827–35 10.1038/nri3769 25421702

[63] Suffia IJ Reckling SK Piccirillo CA Goldszmid RS Belkaid Y. Infected site-restricted Foxp3+ natural regulatory T cells are specific for microbial antigens. J Exp Med (2006) 203:777–88. 10.1084/jem.20052056 16533885 PMC2118233

[64] Suffia I Reckling SK Salay G Belkaid Y. A role for CD103 in the retention of CD4+CD25+ Treg and control of *Leishmania* major infection. J Immunol (2005) 174:5444–55. 10.4049/jimmunol.174.9.5444 15845457

[65] Mendez S Reckling SK Piccirillo CA Sacks D Belkaid Y. Role for CD4(+) CD25(+) regulatory T cells in reactivation of persistent leishmaniasis and control of concomitant immunity. J Exp Med (2004) 200:201–10. 10.1084/jem.20040298 15263027 PMC2212012

[66] Belkaid Y Piccirillo CA Mendez S Shevach EM Sacks DL. CD4+CD25+ regulatory T cells control *Leishmania* major persistence and immunity. Nature (2002) 420:502–7. 10.1038/nature01152 12466842

[67] Nagase H Jones KM Anderson CF Noben-Trauth N. Despite increased CD4+Foxp3+ cells within the infection site, BALB/c IL-4 receptor-deficient mice reveal CD4+Foxp3-negative T cells as a source of IL-10 in *Leishmania* major susceptibility. J Immunol (2007) 179:2435–44. 10.4049/jimmunol.179.4.2435 17675505

[68] Campanelli AP Roselino AM Cavassani KA Pereira MS Mortara RA Brodskyn CI CD4+CD25+ T cells in skin lesions of patients with cutaneous leishmaniasis exhibit phenotypic and functional characteristics of natural regulatory T cells. J Infect Dis (2006) 193:1313–22. 10.1086/502980 16586370

[69] Hoseini SG Javanmard SH Zarkesh SH Khamesipour A Rafiei L Karbalaie K Regulatory T-cell profile in early and late lesions of cutaneous leishmaniasis due to *Leishmania* major. J Res Med Sci (2012) 17:513–8. 23626625 PMC3634286

[70] Bourreau E Ronet C Darcissac E Lise MC Sainte MD Clity E Intralesional regulatory T-cell suppressive function during human acute and chronic cutaneous leishmaniasis due to *Leishmania guyanensis*. Infect Immun (2009) 77:1465–74. 10.1128/IAI.01398-08 19168733 PMC2663152

[71] Bourreau E Ronet C Darsissac E Lise MC Marie DS Clity E In leishmaniasis due to *Leishmania guyanensis* infection, distinct intralesional interleukin-10 and Foxp3 mRNA expression are associated with unresponsiveness to treatment. J Infect Dis (2009) 199:576–9. 10.1086/596508 19125672

[72] Hansen DS Schofield L. Natural regulatory T cells in malaria: host or parasite allies? PLoS Pathog (2010) 6:e1000771. 10.1371/journal.ppat.1000771 20442856 PMC2861684

[73] Minigo G Woodberry T Piera KA Salwati E Tjitra E Kenangalem E Parasite-dependent expansion of TNF receptor II-positive regulatory T cells with enhanced suppressive activity in adults with severe malaria. PLoS Pathog (2009) 5:e1000402. 10.1371/journal.ppat.1000402 19390618 PMC2668192

[74] Torcia MG Santarlasci V Cosmi L Clemente A Maggi L Mangano VD Functional deficit of T regulatory cells in Fulani, an ethnic group with low susceptibility to *Plasmodium falciparum* malaria. Proc Natl Acad Sci U S A (2008) 105:646–51. 10.1073/pnas.0709969105 18174328 PMC2206590

[75] Todryk SM Bejon P Mwangi T Plebanski M Urban B Marsh K Correlation of memory T cell responses against TRAP with protection from clinical malaria, and CD4 CD25 high T cells with susceptibility in Kenyans. PLoS One (2008) 3:e2027. 10.1371/journal.pone.0002027 18446217 PMC2323567

[76] Weinstock JV Elliott DE. Helminth infections decrease host susceptibility to immune-mediated diseases. J Immunol (2014) 193:3239–47. 10.4049/jimmunol.1400927 25240019 PMC4244645

[77] Sawant DV Gravano DM Vogel P Giacomin P Artis D Vignali DA. Regulatory T cells limit induction of protective immunity and promote immune pathology following intestinal helminth infection. J Immunol (2014) 192:2904–12. 10.4049/jimmunol.1202502 24532574 PMC3955731

[78] George PJ Anuradha R Kumaran PP Chandrasekaran V Nutman TB Babu S. Modulation of mycobacterial-specific Th1 and Th17 cells in latent tuberculosis by coincident hookworm infection. J Immunol (2013) 190:5161–8. 10.4049/jimmunol.1203311 23576678 PMC3646958

[79] Babu S Bhat SQ Kumar NP Jayantasri S Rukmani S Kumaran P Human type 1 and 17 responses in latent tuberculosis are modulated by coincident filarial infection through cytotoxic T lymphocyte antigen-4 and programmed death-1. J Infect Dis (2009) 200:288–98. 10.1086/599797 19505258 PMC2997351

[80] Abate E Elias D Getachew A Alemu S Diro E Britton S Effects of albendazole on the clinical outcome and immunological responses in helminth co-infected tuberculosis patients: a double blind randomized clinical trial. Int J Parasitol (2014) 45(2–3):133–40. 10.1016/j.ijpara.2014.09.006 25486494

[81] World Health Organization. Report of the global forum on elimination of leprosy as a public health problem. World Health Organization, Geneva, Switzerland. (2006).

[82] Bobosha K Wilson L van Meijgaarden KE Bekele Y Zewdie M Van Der Ploeg-Van Schip JJ T-cell regulation in lepromatous leprosy. PLoS Negl Trop Dis (2014) 8:e2773. 10.1371/journal.pntd.0002773 24722473 PMC3983090

[83] Fernandes C Goncalves HS Cabral PB Pinto HC Pinto MI Camara LM. Increased frequency of CD4 and CD8 regulatory T cells in individuals under 15 years with multibacillary leprosy. PLoS One (2013) 8:e79072. 10.1371/journal.pone.0079072 24244424 PMC3828331

[84] Palermo ML Pagliari C Trindade MA Yamashitafuji TM Duarte AJ Cacere CR Increased expression of regulatory T cells and down-regulatory molecules in lepromatous leprosy. Am J Trop Med Hyg (2012) 86:878–83. 10.4269/ajtmh.2012.12-0088 22556091 PMC3335697

[85] Saini C Ramesh V Nath I. Increase in TGF-beta secreting CD4(+)CD25(+) FOXP3(+) T regulatory cells in anergic lepromatous leprosy patients. PLoS Negl Trop Dis (2014) 8:e2639. 10.1371/journal.pntd.0002639 24454972 PMC3894184

[86] Kumar S Naqvi RA Ali R Rani R Khanna N Rao DN. CD4+CD25+ T regs with acetylated FoxP3 are associated with immune suppression in human leprosy. Mol Immunol (2013) 56:513–20. 10.1016/j.molimm.2013.04.015 23911408

[87] Callegaro-Filho D Shrestha N Burdick AE Haslett PA. A potential role for complement in immune evasion by *Mycobacterium leprae*. J Drugs Dermatol (2010) 9:1373–82. 21061760

[88] Kraaij MD Savage ND van der Kooij SW Koekkoek K Wang J van den Berg JM Induction of regulatory T cells by macrophages is dependent on production of reactive oxygen species. Proc Natl Acad Sci U S A (2010) 107:17686–91. 10.1073/pnas.1012016107 20861446 PMC2955141

[89] Moura DF de Mattos KA Amadeu TP Andrade PR Sales JS Schmitz V CD163 favors *Mycobacterium leprae* survival and persistence by promoting anti-inflammatory pathways in lepromatous macrophages. Eur J Immunol (2012) 42:2925–36. 10.1002/eji.201142198 22851198

[90] Kumar S Naqvi RA Ali R Rani R Khanna N Rao DN. FoxP3 provides competitive fitness to CD4(+)CD25(+) T cells in leprosy patients via transcriptional regulation. Eur J Immunol (2014) 44:431–9. 10.1002/eji.201343649 24214631

[91] Kumar S Naqvi RA Khanna N Pathak P Rao DN. Th3 immune responses in the progression of leprosy via molecular cross-talks of TGF-beta, CTLA-4 and Cbl-b. Clin Immunol (2011) 141:133–42. 10.1016/j.clim.2011.06.007 21807564

[92] Paolino M Thien CB Gruber T Hinterleitner R Baier G Langdon WY Essential role of E3 ubiquitin ligase activity in Cbl-b-regulated T cell functions. J Immunol (2011) 186:2138–47. 10.4049/jimmunol.1003390 21248250

[93] Abbas AK A network of regulatory pathways in lepromatous leprosy. Clin Immunol (2011) 141:127 10.1016/j.clim.2011.08.007 21903479

[94] Shafiani S Tucker-Heard G Kariyone A Takatsu K Urdahl KB. Pathogen-specific regulatory T cells delay the arrival of effector T cells in the lung during early tuberculosis. J Exp Med (2010) 207:1409–20. 10.1084/jem.20091885 20547826 PMC2901066

[95] Scott-Browne JP Shafiani S Tucker-Heard G Ishida-Tsubota K Fontenot JD Rudensky AY Expansion and function of Foxp3-expressing T regulatory cells during tuberculosis. J Exp Med (2007) 204:2159–69. 10.1084/jem.20062105 17709423 PMC2118702

[96] Kursar M Koch M Mittrucker HW Nouailles G Bonhagen K Kamradt T Cutting edge: regulatory T cells prevent efficient clearance of *Mycobacterium tuberculosis*. J Immunol (2007) 178:2661–5. 10.4049/jimmunol.178.5.2661 17312107

[97] Shafiani S Dinh C Ertelt JM Moguche AO Siddiqui I Smigiel KS Pathogen-specific Treg cells expand early during *Mycobacterium tuberculosis* infection but are later eliminated in response to interleukin-12. Immunity (2013) 38:1261–70. 10.1016/j.immuni.2013.06.003 23791647 PMC3827956

[98] Ottenhoff TH. New pathways of protective and pathological host defense to mycobacteria. Trends Microbiol (2012) 20(9):419–28. 10.1016/j.tim.2012.06.002 22784857

[99] Cooper AM Cell-mediated immune responses in tuberculosis. Annu Rev Immunol (2009) 27:393–422 10.1146/annurev.immunol.021908.132703 19302046 PMC4298253

[100] Ozeki Y Sugawara I Udagawa T Aoki T Osada-Oka M Tateishi Y Transient role of CD4+CD25+ regulatory T cells in mycobacterial infection in mice. Int Immunol (2010) 22:179–89. 10.1093/intimm/dxp126 20139174

[101] Chen CY Huang D Yao S Halliday L Zeng G Wang RC IL-2 simultaneously expands Foxp3+ T regulatory and T effector cells and confers resistance to severe tuberculosis (TB): implicative Treg-T effector cooperation in immunity to TB. J Immunol (2012) 188:4278–88. 10.4049/jimmunol.1101291 22474020 PMC3412415

[102] Gratz IK Campbell DJ. Organ-specific and memory treg cells: specificity, development, function, and maintenance. Front Immunol (2014) 5:333. 10.3389/fimmu.2014.00333 25076948 PMC4098124

[103] Guyot-Revol V Innes JA Hackforth S Hinks T Lalvani A. Regulatory T cells are expanded in blood and disease sites in patients with tuberculosis. Am J Respir Crit Care Med (2006) 173:803–10. 10.1164/rccm.200508-1294OC 16339919

[104] Ribeiro-Rodrigues R Resende CT Rojas R Toossi Z Dietze R Boom WH A role for CD4+CD25+ T cells in regulation of the immune response during human tuberculosis. Clin Exp Immunol (2006) 144:25–34. 10.1111/j.1365-2249.2006.03027.x 16542361 PMC1809641

[105] Chiacchio T Casetti R Butera O Vanini V Carrara S Girardi E Characterization of regulatory T cells identified as CD4(+)CD25(high)CD39(+) in patients with active tuberculosis. Clin Exp Immunol (2009) 156:463–70. 10.1111/j.1365-2249.2009.03908.x 19438599 PMC2691975

[106] Singh A Dey AB Mohan A Sharma PK Mitra DK. Foxp3+ regulatory T cells among tuberculosis patients: impact on prognosis and restoration of antigen specific IFN-gamma producing T cells. PLoS One (2012) 7:e44728. 10.1371/journal.pone.0044728 23028594 PMC3446959

[107] Semple PL Binder AB Davids M Maredza A van Zyl-Smit RN Dheda K. Regulatory T cells attenuate mycobacterial stasis in alveolar and blood-derived macrophages from patients with tuberculosis. Am J Respir Crit Care Med (2013) 187:1249–58. 10.1164/rccm.201210-1934OC 23590266

[108] Burl S Hill PC Jeffries DJ Holland MJ Fox A Lugos MD FOXP3 gene expression in a tuberculosis case contact study. Clin Exp Immunol (2007) 149:117–22. 10.1111/j.1365-2249.2007.03399.x 17465993 PMC1942016

[109] Joosten SA van Meijgaarden KE Savage ND de Boer T Triebel F van der Wal A Identification of a human CD8+ regulatory T cell subset that mediates suppression through the chemokine CC chemokine ligand 4. Proc Natl Acad Sci U S A (2007) 104:8029–34. 10.1073/pnas.0702257104 17483450 PMC1876566

[110] Joosten SA van Meijgaarden KE van Weeren PC Kazi F Geluk A Savage ND *Mycobacterium tuberculosis* peptides presented by HLA-E molecules are targets for human CD8 T-cells with cytotoxic as well as regulatory activity. PLoS Pathog (2010) 6:e1000782. 10.1371/journal.ppat.1000782 20195504 PMC2829052

[111] Meijgaarden KE Haks MC Caccamo N Dieli F Ottenhoff TH Joosten SA. Human CD8+ T-cells recognizing peptides from *Mycobacterium tuberculosis* (Mtb) presented by HLA-E have an unorthodox Th2-like, multifunctional, Mtb inhibitory phenotype and represent a novel human T-cell subset. PLoS Pathog (2015) 11(3):e1004671. 10.1371/journal.ppat.1004671 25803478 PMC4372528

[112] Lim HJ Park JS Cho YJ Yoon HI Park KU Lee CT CD4(+)FoxP3(+) T regulatory cells in drug-susceptible and multidrug-resistant tuberculosis. Tuberculosis (Edinb) (2013) 93:523–8. 10.1016/j.tube.2013.06.001 23810735

[113] Pinheiro RO de Oliveira EB Dos SG Sperandio da Silva GM de Andrade Silva BJ Teles RM Different immunosuppressive mechanisms in multi-drug-resistant tuberculosis and non-tuberculous mycobacteria patients. Clin Exp Immunol (2013) 171:210–9. 10.1111/cei.12007 23286948 PMC3573292

[114] Marin ND Paris SC Velez VM Rojas CA Rojas M Garcia LF. Regulatory T cell frequency and modulation of IFN-gamma and IL-17 in active and latent tuberculosis. Tuberculosis (Edinb) (2010) 90:252–61. 10.1016/j.tube.2010.05.003 20594914

[115] Kim K Perera R Tan DB Fernandez S Seddiki N Waring J Circulating mycobacterial-reactive CD4+ T cells with an immunosuppressive phenotype are higher in active tuberculosis than latent tuberculosis infection. Tuberculosis (Edinb) (2014) 94:494–501. 10.1016/j.tube.2014.07.002 25095750

[116] Place S Verscheure V de San N Hougardy JM Schepers K Dirix V Heparin-binding, hemagglutinin-specific IFN-gamma synthesis at the site of infection during active tuberculosis in humans. Am J Respir Crit Care Med (2010) 182:848–54. 10.1164/rccm.201001-0083OC 20508213

[117] Rozot V Vigano S Mazza-Stalder J Idrizi E Day CL Perreau M *Mycobacterium tuberculosis*-specific CD8+ T cells are functionally and phenotypically different between latent infection and active disease. Eur J Immunol (2013) 43:1568–77. 10.1002/eji.201243262 23456989 PMC6535091

[118] Lewinsohn DA Heinzel AS Gardner JM Zhu L Alderson MR Lewinsohn DM. *Mycobacterium tuberculosis*-specific CD8+ T cells preferentially recognize heavily infected cells. Am J Respir Crit Care Med (2003) 168:1346–52. 10.1164/rccm.200306-837OC 12969871

[119] Silva BD Trentini MM da Costa AC Kipnis A Junqueira-Kipnis AP. Different phenotypes of CD8+ T cells associated with bacterial load in active tuberculosis. Immunol Lett (2014) 160:23–32. 10.1016/j.imlet.2014.03.009 24694750

[120] Cyktor JC Carruthers B Beamer GL Turner J. Clonal expansions of CD8+ T cells with IL-10 secreting capacity occur during chronic *Mycobacterium tuberculosis* infection. PLoS One (2013) 8:e58612. 10.1371/journal.pone.0058612 23472214 PMC3589362

[121] Garcia Jacobo RE Serrano CJ Enciso Moreno JA Gaspar RO Trujillo Ochoa JL Uresti Rivera EE Analysis of Th1, Th17 and regulatory T cells in tuberculosis case contacts. Cell Immunol (2014) 289:167–73. 10.1016/j.cellimm.2014.03.010 24841855

[122] Hopewell PC Clinical Features of Tuberculosis. In: Kaufmann SH van Helden P, editors. Handbook of Tuberculosis: Clinics, Diagnostics, Therapy and Epidemiology. Weinheim: Wiley-VCH Verlag GmbH & Co (2008). p. 89–114.

[123] Sharma PK Saha PK Singh A Sharma SK Ghosh B Mitra DK. FoxP3+ regulatory T cells suppress effector T-cell function at pathologic site in miliary tuberculosis. Am J Respir Crit Care Med (2009) 179:1061–70. 10.1164/rccm.200804-529OC 19246720

[124] de Almeida AS Fiske CT Sterling TR Kalams SA. Increased frequency of regulatory T cells and T lymphocyte activation in persons with previously treated extrapulmonary tuberculosis. Clin Vaccine Immunol (2012) 19:45–52. 10.1128/CVI.05263-11 22038848 PMC3255960

[125] Wu C Zhou Q Qin XJ Qin SM Shi HZ. CCL22 is involved in the recruitment of CD4+CD25 high T cells into tuberculous pleural effusions. Respirology (2010) 15:522–9. 10.1111/j.1440-1843.2010.01719.x 20337996

[126] Geffner L Basile JI Yokobori N Sabio YG Musella R Castagnino J CD4(+) CD25(high) forkhead box protein 3(+) regulatory T lymphocytes suppress interferon-gamma and CD107 expression in CD4(+) and CD8(+) T cells from tuberculous pleural effusions. Clin Exp Immunol (2014) 175:235–45. 10.1111/cei.12227 24134738 PMC3892415

[127] Ye ZJ Zhou Q Du RH Li X Huang B Shi HZ. Imbalance of Th17 cells and regulatory T cells in tuberculous pleural effusion. Clin Vaccine Immunol (2011) 18:1608–15. 10.1128/CVI.05214-11 21813663 PMC3187020

[128] Yuan ML Tong ZH Jin XG Zhang JC Wang XJ Ma WL Regulation of CD4(+) T cells by pleural mesothelial cells via adhesion molecule-dependent mechanisms in tuberculous pleurisy. PLoS One (2013) 8:e74624. 10.1371/journal.pone.0074624 24069325 PMC3777994

[129] Rahman S Gudetta B Fink J Granath A Ashenafi S Aseffa A Compartmentalization of immune responses in human tuberculosis: few CD8+ effector T cells but elevated levels of FoxP3+ regulatory t cells in the granulomatous lesions. Am J Pathol (2009) 174:2211–24. 10.2353/ajpath.2009.080941 19435796 PMC2684186

[130] Saenz B Hernandez-Pando R Fragoso G Bottasso O Cardenas G. The dual face of central nervous system tuberculosis: a new Janus Bifrons? Tuberculosis (Edinb) (2013) 93:130–5. 10.1016/j.tube.2012.11.011 23305698

[131] Tan DB Yong YK Tan HY Kamarulzaman A Tan LH Lim A Immunological profiles of immune restoration disease presenting as mycobacterial lymphadenitis and cryptococcal meningitis. HIV Med (2008) 9:307–16. 10.1111/j.1468-1293.2008.00565.x 18400078

[132] Zaidi I Peterson K Jeffries D Whittle H de ST Rowland-Jones S Immune reconstitution inflammatory syndrome and the influence of T regulatory cells: a cohort study in the Gambia. PLoS One (2012) 7:e39213. 10.1371/journal.pone.0039213 22745716 PMC3380048

[133] Haddow LJ Dibben O Moosa MY Borrow P Easterbrook PJ. Circulating inflammatory biomarkers can predict and characterize tuberculosis-associated immune reconstitution inflammatory syndrome. AIDS (2011) 25:1163–74. 10.1097/QAD.0b013e3283477d67 21505297

[134] Seddiki N Sasson SC Santner-Nanan B Munier M van BD Ip S Proliferation of weakly suppressive regulatory CD4+ T cells is associated with over-active CD4+ T-cell responses in HIV-positive patients with mycobacterial immune restoration disease. Eur J Immunol (2009) 39:391–403. 10.1002/eji.200838630 19180462

[135] Boussiotis VA Tsai EY Yunis EJ Thim S Delgado JC Dascher CC IL-10-producing T cells suppress immune responses in anergic tuberculosis patients. J Clin Invest (2000) 105:1317–25. 10.1172/JCI9918 10792007 PMC315449

[136] Delgado JC Tsai EY Thim S Baena A Boussiotis VA Reynes JM Antigen-specific and persistent tuberculin anergy in a cohort of pulmonary tuberculosis patients from rural Cambodia. Proc Natl Acad Sci U S A (2002) 99:7576–81. 10.1073/pnas.062056099 12032325 PMC124289

[137] Ranjbar S Ly N Thim S Reynes JM Goldfeld AE. *Mycobacterium tuberculosis* recall antigens suppress HIV-1 replication in anergic donor cells via CD8+ T cell expansion and increased IL-10 levels. J Immunol (2004) 172:1953–9. 10.4049/jimmunol.172.3.1953 14734781

[138] Hougardy JM Place S Hildebrand M Drowart A Debrie AS Locht C Regulatory T cells depress immune responses to protective antigens in active tuberculosis. Am J Respir Crit Care Med (2007) 176:409–16. 10.1164/rccm.200701-084OC 17541018

[139] de Cassan SC Pathan AA Sander CR Minassian A Rowland R Hill AV Investigating the induction of vaccine-induced Th17 and regulatory T cells in healthy, *Mycobacterium bovis* BCG-immunized adults vaccinated with a new tuberculosis vaccine, MVA85A. Clin Vaccine Immunol (2010) 17:1066–73. 10.1128/CVI.00047-10 20484570 PMC2897259

[140] Griffiths KL Pathan AA Minassian AM Sander CR Beveridge NE Hill AV Th1/Th17 cell induction and corresponding reduction in ATP consumption following vaccination with the novel *Mycobacterium tuberculosis* vaccine MVA85A. PLoS One (2011) 6:e23463. 10.1371/journal.pone.0023463 21887254 PMC3162567

[141] Pinheiro MB Antonelli LR Sathler-Avelar R Vitelli-Avelar DM Spindola-de-Miranda S Guimaraes TM CD4-CD8-alphabeta and gammadelta T cells display inflammatory and regulatory potentials during human tuberculosis. PLoS One (2012) 7:e50923. 10.1371/journal.pone.0050923 23239994 PMC3519797

[142] Jackson-Sillah D Cliff JM Mensah GI Dickson E Sowah S Tetteh JK Recombinant ESAT-6-CFP10 fusion protein induction of Th1/Th2 cytokines and FoxP3 expressing Treg cells in pulmonary TB. PLoS One (2013) 8:e68121. 10.1371/journal.pone.0068121 23826366 PMC3694917

[143] He XY Xiao L Chen HB Hao J Li J Wang YJ T regulatory cells and Th1/Th2 cytokines in peripheral blood from tuberculosis patients. Eur J Clin Microbiol Infect Dis (2010) 29:643–50. 10.1007/s10096-010-0908-0 20306324

[144] Feruglio SL Tonby K Kvale D Dyrhol-Riise AM. Early dynamics of T helper cell cytokines and T regulatory cells in response to treatment of active *Mycobacterium tuberculosis* infection. Clin Exp Immunol (2014) 179(3):454–65. 10.1111/cei.12468 25313008 PMC4337678

[145] Sprengers D Stoop JN Binda RS Kusters JG Haagmans BL Carotenuto P Induction of regulatory T-cells and interleukin-10-producing cells in non-responders to pegylated interferon-alpha therapy for chronic hepatitis B. Antivir Ther (2007) 12:1087–96. 18018767

[146] Ndure J Flanagan KL. Targeting regulatory T cells to improve vaccine immunogenicity in early life. Front Microbiol (2014) 5:477. 10.3389/fmicb.2014.00477 25309517 PMC4161046

[147] Ha TY Waksman BH Treffers HP. The thymic suppressor cell. I. Separation of subpopulations with suppressor activity. J Exp Med (1974) 139:13–23. 10.1084/jem.139.1.13 4128445 PMC2139511

[148] Marchant A Goetghebuer T Ota MO Wolfe I Ceesay SJ de Groote D Newborns develop a Th1-type immune response to *Mycobacterium bovis Bacillus* Calmette-Guerin vaccination. J Immunol (1999) 163:2249–55. 10438968

[149] Hussey GD Watkins ML Goddard EA Gottschalk S Hughes EJ Iloni K Neonatal mycobacterial specific cytotoxic T-lymphocyte and cytokine profiles in response to distinct BCG vaccination strategies. Immunology (2002) 105:314–24. 10.1046/j.1365-2567.2002.01366.x 11918693 PMC1782661

[150] Kaufmann SH. Tuberculosis vaccines: time to think about the next generation. Semin Immunol (2013) 25:172–81. 10.1016/j.smim.2013.04.006 23706597

[151] Hanekom WA. The immune response to BCG vaccination of newborns. Ann N Y Acad Sci (2005) 1062:69–78. 10.1196/annals.1358.010 16461790

[152] Akkoc T Aydogan M Yildiz A Karakoc-Aydiner E Eifan A Keles S Neonatal BCG vaccination induces IL-10 production by CD4+ CD25+ T cells. Pediatr Allergy Immunol (2010) 21:1059–63. 10.1111/j.1399-3038.2010.01051.x 20977501

[153] Li L Qiao D Zhang X Liu Z Wu C. The immune responses of central and effector memory BCG-specific CD4+ T cells in BCG-vaccinated PPD+ donors were modulated by Treg cells. Immunobiology (2011) 216:477–84. 10.1016/j.imbio.2010.09.003 20950889

[154] Boer MC van Meijgaarden KE Joosten SA Ottenhoff TH. CD8+ regulatory T cells, and not CD4+ T cells, dominate suppressive phenotype and function after in vitro live *Mycobacterium bovis*-BCG activation of human cells. PLoS One (2014) 9:e94192. 10.1371/journal.pone.0094192 24714620 PMC3979753

[155] Boer MC van Meijgaarden KE Bastid J Ottenhoff TH Joosten SA. CD39 is involved in mediating suppression by *Mycobacterium bovis* BCG-activated human CD8(+) CD39(+) regulatory T cells. Eur J Immunol (2013) 43:1925–32. 10.1002/eji.201243286 23606272

[156] Day CL Tameris M Mansoor N van RM de KM Geldenhuys H Induction and regulation of T-cell immunity by the novel tuberculosis vaccine M72/AS01 in South African adults. Am J Respir Crit Care Med (2013) 188:492–502. 10.1164/rccm.201208-1385OC 23306546 PMC3778736

[157] Matsumiya M Stylianou E Griffiths K Lang Z Meyer J Harris SA Roles for Treg expansion and HMGB1 signaling through the TLR1-2-6 axis in determining the magnitude of the antigen-specific immune response to MVA85A. PLoS One (2013) 8:e67922. 10.1371/journal.pone.0067922 23844129 PMC3700883

[158] Matsumiya M Harris SA Satti I Stockdale L Tanner R O’Shea MK Inflammatory and myeloid-associated gene expression before and one day after infant vaccination with MVA85A correlates with induction of a T cell response. BMC Infect Dis (2014) 14:314. 10.1186/1471-2334-14-314 24912498 PMC4061512

[159] Andersen P Doherty TM. The success and failure of BCG – implications for a novel tuberculosis vaccine. Nat Rev Microbiol (2005) 3:656–62. 10.1038/nrmicro1211 16012514

[160] Coleman MM Keane J Mills KH Editorial: Tregs and BCG – dangerous liaisons in TB. J Leukoc Biol (2010) 88:1067–9 10.1189/jlb.0710419 21123295

[161] Ho P Wei X Seah GT. Regulatory T cells induced by *Mycobacterium chelonae* sensitization influence murine responses to bacille Calmette-Guerin. J Leukoc Biol (2010) 88:1073–80. 10.1189/jlb.0809582 20651297

[162] Beverley P Ronan E Lee L Arnold I Bolinger B Powrie F Environmental effects on protection against *Mycobacterium tuberculosis* after immunization with Ad85A. Vaccine (2013) 31:1086–93. 10.1016/j.vaccine.2012.12.024 23262169 PMC3566543

[163] Perry S Hussain R Parsonnet J. The impact of mucosal infections on acquisition and progression of tuberculosis. Mucosal Immunol (2011) 4:246–51. 10.1038/mi.2011.11 21412228 PMC5480373

[164] Elias D Britton S Aseffa A Engers H Akuffo H. Poor immunogenicity of BCG in helminth infected population is associated with increased in vitro TGF-beta production. Vaccine (2008) 26:3897–902. 10.1016/j.vaccine.2008.04.083 18554755

[165] Lacan G Dang H Middleton B Horwitz MA Tian J Melega WP *Bacillus* Calmette-Guerin vaccine-mediated neuroprotection is associated with regulatory T-cell induction in the 1-methyl-4-phenyl-1,2,3,6-tetrahydropyridine mouse model of Parkinson’s disease. J Neurosci Res (2013) 91:1292–302. 10.1002/jnr.23253 23907992 PMC5800426

[166] Lee J Reinke EK Zozulya AL Sandor M Fabry Z. *Mycobacterium bovis* bacille Calmette-Guerin infection in the CNS suppresses experimental autoimmune encephalomyelitis and Th17 responses in an IFN-gamma-independent manner. J Immunol (2008) 181:6201–12. 10.4049/jimmunol.181.9.6201 18941210 PMC2735452

[167] Kim YJ Kim HJ Kang MJ Yu HS Seo JH Kim HY *Bacillus* Calmette-Guerin suppresses asthmatic responses via CD4(+)CD25(+) regulatory T cells and dendritic cells. Allergy Asthma Immunol Res (2014) 6:201–7. 10.4168/aair.2014.6.3.201 24843794 PMC4021237

[168] Lagranderie M Kluge C Kiefer-Biasizzo H Abolhassani M Nahori MA Fitting C *Mycobacterium bovis Bacillus* Calmette-Guerin killed by extended freeze-drying reduces colitis in mice. Gastroenterology (2011) 141(642–52):652. 10.1053/j.gastro.2011.05.002 21683076

[169] Andrieu JM Chen S Lai C Guo W Lu W. Mucosal SIV vaccines comprising inactivated virus particles and bacterial adjuvants induce CD8(+) T-regulatory cells that suppress SIV-positive CD4(+) T-cell activation and prevent SIV infection in the macaque model. Front Immunol (2014) 5:297. 10.3389/fimmu.2014.00297 25071760 PMC4074992

[170] Bhattacharya D Dwivedi VP Maiga M Maiga M Van KL Bishai WR Small molecule-directed immunotherapy against recurrent infection by *Mycobacterium tuberculosis*. J Biol Chem (2014) 289:16508–15. 10.1074/jbc.M114.558098 24711459 PMC4047417

[171] Bhattacharya D Dwivedi VP Kumar S Reddy MC Kaer LV Moodley P Simultaneous inhibition of T helper 2 and T regulatory cell differentiation by small molecules enhances *Bacillus* Calmette-Guerin vaccine efficacy against tuberculosis. J Biol Chem (2014) 289(48):33404–11. 10.1074/jbc.M114.600452 25315774 PMC4246096

[172] Pitt JM Stavropoulos E Redford PS Beebe AM Bancroft GJ Young DB Blockade of IL-10 signaling during *Bacillus* Calmette-Guerin vaccination enhances and sustains Th1, Th17, and innate lymphoid IFN-gamma and IL-17 responses and increases protection to *Mycobacterium tuberculosis* infection. J Immunol (2012) 189:4079–87. 10.4049/jimmunol.1201061 22972927 PMC3467194

[173] Dhiman R Periasamy S Barnes PF Jaiswal AG Paidipally P Barnes AB NK1.1+ cells and IL-22 regulate vaccine-induced protective immunity against challenge with *Mycobacterium tuberculosis*. J Immunol (2012) 189:897–905. 10.4049/jimmunol.1102833 22711885 PMC3392427

[174] Jaron B Maranghi E Leclerc C Majlessi L. Effect of attenuation of Treg during BCG immunization on anti-mycobacterial Th1 responses and protection against *Mycobacterium tuberculosis*. PLoS One (2008) 3:e2833. 10.1371/journal.pone.0002833 18665224 PMC2475666

[175] Berod L Stuve P Varela F Behrends J Swallow M Kruse F Rapid rebound of the Treg compartment in DEREG mice limits the impact of Treg depletion on mycobacterial burden, but prevents autoimmunity. PLoS One (2014) 9:e102804. 10.1371/journal.pone.0102804 25050936 PMC4106855

[176] Bayry J Tchilian EZ Davies MN Forbes EK Draper SJ Kaveri SV In silico identified CCR4 antagonists target regulatory T cells and exert adjuvant activity in vaccination. Proc Natl Acad Sci U S A (2008) 105:10221–6. 10.1073/pnas.0803453105 18621704 PMC2481334

[177] Mills KH. Designer adjuvants for enhancing the efficacy of infectious disease and cancer vaccines based on suppression of regulatory T cell induction. Immunol Lett (2009) 122:108–11. 10.1016/j.imlet.2008.11.007 19100777

